# Infliximab for medical induction of remission in Crohn's disease

**DOI:** 10.1002/14651858.CD012623.pub2

**Published:** 2023-11-20

**Authors:** Morris Gordon, Vassiliki Sinopoulou, Anthony K Akobeng, Shellie J Radford, Mohsen Eldragini, Ana-Maria Darie, Gordon William Moran

**Affiliations:** School of MedicineUniversity of Central LancashirePrestonUK; Pediatric GastroenterologySidra MedicineDohaQatar; NIHR Nottingham Biomedical Research Centre – Gastrointestinal and Liver disorders themeNottingham University Hospitals NHS TrustNottinghamUK; National Institute of Health Research Nottingham Biomedical Research CentreUniversity of Nottingham and Nottingham University HospitalsNottinghamUK

**Keywords:** Adult, Aged, Humans, Middle Aged, Antimetabolites, Biosimilar Pharmaceuticals, Crohn Disease, Crohn Disease/drug therapy, Infliximab, Infliximab/therapeutic use, Purines, Remission Induction

## Abstract

**Background:**

Infliximab is a monoclonal antibody that binds and neutralises tumour necrosis factor‐alpha (TNF‐α), which is present in high levels in the blood serum, mucosa and stool of people with Crohn's disease.

**Objectives:**

To evaluate the benefits and harms of infliximab alone or in combination with another agent for induction of remission in Crohn's disease compared to placebo or active medical therapies.

**Search methods:**

On 31 August 2021 and 4 March 2023, we searched CENTRAL, MEDLINE, Embase, ClinicalTrials.gov and World Health Organization ICTRP.

**Selection criteria:**

Randomised control trials (RCTs) comparing infliximab alone or in combination with another agent to placebo or another active comparator in adults with active Crohn's disease.

**Data collection and analysis:**

Pairs of review authors independently selected studies and conducted data extraction and risk of bias assessment. We expressed outcomes as risk ratios (RR) and mean differences (MD) with 95% confidence intervals (CI). We assessed the certainty of the evidence using GRADE.

Our primary outcomes were clinical remission, clinical response and withdrawals due to adverse events. Our secondary outcomes were endoscopic remission, histological remission, endoscopic response, and serious and total adverse events.

**Main results:**

The search identified 10 RCTs with 1101 participants. They were conducted between 1999 and 2019, and 7/10 RCTs included biologically naive participants. All but one RCT, which did not provide information, were multicentre and funded by pharmaceutical companies, and their authors declared conflicts. The age of the participants ranged from 26 to 65 years. Results were based on one study unless otherwise stated.

Infliximab 5 mg/kg to 10 mg/kg may be more effective than placebo at week four for clinical remission (30/55 versus 3/25; RR 4.55, 95% CI 1.53 to 13.50; number needed to treat for an additional beneficial outcome (NNTB) 3) and response (36/55 versus 4/25; RR 4.09, 95% CI 1.63 to 10.25, NNTB 3). The evidence was low certainty. The study did not report withdrawals due to adverse events.

We could not draw conclusions on the effects of infliximab 5 mg/kg to 10 mg/kg compared to placebo for fistulating participants for clinical remission (29/63 versus 4/31; RR 3.57, 95% CI 1.38 to 9.25; NNTB 4), response (48/106 versus 15/75; RR 1.94, 95% CI 1.10 to 3.41; NNTB 6; 2 studies) or withdrawals due to adverse events (2/63 versus 0/31; RR 2.50, 95% CI 0.12 to 50.54). The evidence was very low certainty.

Infliximab used in combination with purine analogues is probably more effective than purine analogues alone for clinical remission at weeks 24 to 26 (182/301 versus 95/302; RR 1.92, 95% CI 1.59 to 2.32, NNTB 4; 4 studies; moderate‐certainty evidence) and clinical response at week 26 (107/177 versus 66/178; RR 1.64, 95% CI 1.31 to 2.05; NNTB 5; 2 studies; moderate‐certainty evidence). There may be little or no difference in withdrawals due to adverse events at week 26 (62/302 versus 53/301; RR 0.87, 95% CI 0.63 to 1.21; 4 studies; low‐certainty evidence).

Infliximab alone may be more effective than purine analogues alone at week 26 for clinical remission (85/177 versus 57/178; RR 1.50, 95% CI 1.15 to 1.95; NNTB 7; 2 studies) and response (94/177 versus 66/178; RR 1.44, 95% CI 1.13 to 1.82; NNTB 7; 2 studies). There may be little or no difference in withdrawals due to adverse events (30/177 versus 43/178; RR 0.70, 95% CI 0.46 to 1.06; 4 studies). The evidence was low certainty.

We could not draw any conclusions on the effects of infliximab 5 mg/kg compared to 10 mg/kg for clinical remission (19/27 versus 11/28; RR 1.79, 95% CI 1.06 to 3.02) and response (22/27 versus 24/28; RR 1.63, 95% CI 1.08 to 2.46). The evidence was very low certainty. Withdrawals due to adverse events were not reported.

We could not draw any conclusions on the effects of infliximab 5 mg/kg compared to 10 mg/kg in an exclusively fistulating population for clinical remission (17/31 versus 12/32; RR 1.46, 95% CI 0.84 to 2.53), response (21/31 versus 18/32; RR 1.20, 95% CI 0.82 to 1.78), or withdrawals due to adverse events (1/31 versus 1/32; RR 1.03, 95% CI 0.07 to 15.79). The evidence was very low certainty.

We could not draw any conclusions on the effects of infliximab 5 mg/kg compared to 20 mg/kg for clinical remission (19/27 versus 11/28; RR 1.79, 95% CI 1.06 to 3.02) or response (22/27 versus 18/28; RR 1.27, 95% CI 0.91 to 1.76). The evidence was very low certainty. Withdrawals due to adverse events were not reported.

We could not draw any conclusions on the effects of infliximab 10 mg/kg compared to 20 mg/kg for clinical remission (11/28 versus 11/28; RR 1.00, 95% CI 0.52 to 1.92) or response (14/28 versus 18/28; RR 0.78, 95% CI 0.49 to 1.23). The evidence was very low certainty. Withdrawals due to adverse events were not reported.

There may be little or no difference between infliximab and a CT‐P13 biosimilar at week six for clinical remission (47/109 versus 49/111; RR 0.98, 95% CI 0.72 to 1.32), response (67/109 versus 70/111; RR 0.97, 95% CI 0.79 to 1.20) and withdrawals due to adverse events (21/109 versus 17/111; RR 1.26, 95% CI 0.70 to 2.25). The evidence was low certainty.

**Authors' conclusions:**

Infliximab in combination with purine analogues is probably more effective than purine analogues alone in inducing clinical remission and clinical response. Infliximab alone may be more effective in inducing clinical remission and response than purine analogues alone or placebo. Infliximab may be similar in efficacy to a CT‐P13 biosimilar and there may be little or no difference in withdrawals due to adverse events.

We were unable to draw meaningful conclusions as to whether infliximab alone is effective when used for exclusively fistulating populations.

There was evidence that there may be little or no difference in withdrawal due to adverse events between infliximab plus purines compared with purines alone, as well as infliximab alone compared with purines alone. Meaningful conclusions cannot be drawn on all other outcomes related to adverse events due to very low certainty evidence.

## Plain language summary

**Infliximab for the treatment of active Crohn's disease**

**Key messages**

– Infliximab used with purine analogues (azathioprine or 6‐mercaptopurine) is probably more effective than purine analogues alone at getting Crohn's into remission. It may also be better at improving symptoms. The two treatments may be similar in terms of safety.

– Infliximab alone may be more effective than purine analogues alone for getting Crohn's into remission and improving symptoms. The two treatments may be similar in terms of safety.

– Infliximab may be as effective as the biosimilar at getting Crohn's into remission and improving symptoms. The two treatments may be similar in terms of safety. An infliximab biosimilar is a biological medicine (contains substances that have been created by using living cells or organisms) that is highly similar to the original brand of infliximab.

**What is Crohn's disease?**

Crohn's disease is a life‐long inflammatory disease that can affect any part of the gut. Common symptoms include bloody poo, diarrhoea, stomach ache, fever, weight loss and fatigue. We do not know exactly what causes Crohn's, but it is probably a mix of genes, problems with the immune system (which defends the body against infection), bacteria in the gut and something in the environment.

There is no known cure for Crohn's, but the symptoms are usually managed with medicines, such as corticosteroids and immune system medications, and sometimes surgery. Infliximab is a type of Crohn's medicine called a biological medicine.

Most people with Crohn's have times when they have symptoms and other times when their symptoms are under control. When they have symptoms, it is called active disease. When their symptoms are under control, it is called remission.

**What did we want to find out?**

We wanted to find out how infliximab compares to other medicines or dummy treatment (placebo) for getting Crohn's into remission or improving symptoms. We also wanted to find out how safe it is compared to other medicines.

**What did we do?**

We searched for randomised controlled trials (studies where people are assigned to one of two or more treatment groups using a random method) comparing infliximab with any other medical treatment in adults with Crohn's disease.

**What did we find?**

We found 10 studies including 1101 participants. They looked at:

– infliximab compared to placebo (one study);

– infliximab plus purine analogues compared to purine analogues alone (four studies);

– infliximab compared to purine analogues (medicines called azathioprine or 6‐mercaptopurine) (two studies);

– infliximab compared to a biosimilar (one study);

– different doses of infliximab compared to each other (two studies).

Two studies looked at people with Crohn's disease who had fistulas. A fistula is a narrow tunnel that can develop between your gut and your skin or another organ, such as your bladder.

**Main results**

Infliximab plus purine analogues (azathioprine or 6‐mercaptopurine) is probably more effective than purine analogues alone at getting Crohn's into remission after 24 to 26 weeks of treatment. It may also be better at improving symptoms. The two treatments may be similar in terms of side effects.

Infliximab alone may be more effective than purine analogues alone for getting Crohn's into remission and improving symptoms after 26 weeks of treatment. The two treatments may be similar in terms of side effects.

Infliximab may be as effective as the biosimilar at getting Crohn's into remission and improving symptoms after six weeks of treatment. The two treatments may be similar in terms of side effects.

There is not enough evidence to compare the other treatments we looked at in this review.

**What are the limitations of the evidence?**

The evidence is mostly of low and very low quality. This is because of problems with the way the trials were carried out, the small number of people who took part and problems with how the results were reported.

**How up‐to‐date is this review?**

This review is up‐to‐date to 4 March 2023.

## Summary of findings

**Summary of findings 1:** Infliximab 5–10 mg/kg compared to placebo

**Infliximab compared to placebo**						
**Patient or population:** active Crohn's disease **Setting:** hospitals and tertiary centres (Amsterdam, Belgium, the Netherlands, UK, USA) **Intervention:** infliximab **Comparison:** placebo						
**Outcomes**	Anticipated absolute effects^*^ (95% CI)		**Relative effect (95% CI)**	**№ of participants (studies)**	**Certainty of the evidence (GRADE)**	**Comments**
	**Risk with placebo**	**Risk with infliximab**				
**Clinical remission** defined as CDAI < 150 at week 4	120 per 1000	546 per 1000 (184 to 1000)	**RR 4.55** (1.53 to 13.50)	80 (1 study)	⊕⊕⊝⊝ **Low**^a^	—
**Clinical response** defined as improvement in the scores on the CDAI score ≥ 70 at week 4	160 per 1000	654 per 1000 (260 to 1000)	**RR 4.09** (1.63 to 10.25)	80 (1 studies)	⊕⊕⊝⊝ **Low**^a^	—
**Withdrawals due to adverse events**	—		—	—	—	—
*The risk in the intervention group (and its 95% confidence interval) is based on the assumed risk in the comparison group and the **relative effect** of the intervention (and its 95% CI). **CDAI:** Crohn's Disease Activity Index; **CI:** confidence interval; **RR:** risk ratio.						
**GRADE Working Group grades of evidence** **High certainty:** we are very confident that the true effect lies close to that of the estimate of the effect. **Moderate certainty:** we are moderately confident in the effect estimate: the true effect is likely to be close to the estimate of the effect, but there is a possibility that it is substantially different. **Low certainty:** our confidence in the effect estimate is limited: the true effect may be substantially different from the estimate of the effect. **Very low certainty:** we have very little confidence in the effect estimate: the true effect is likely to be substantially different from the estimate of effect.						

^a^Downgraded one level due to serious concerns with risk of bias (selective reporting and unclear randomisation), and one level due to serious concerns with imprecision due to low event numbers.

**Summary of findings 2:** Infliximab 5–10 mg/kg compared to placebo for exclusively fistulating population

**Infliximab compared to placebo for exclusively fistulating population**						
**Patient or population:** active Crohn's disease **Setting:** not reported (multiple countries) **Intervention:** infliximab (combined 5 and 10 mg/kg dosages) **Comparison:** placebo						
**Outcomes**	Anticipated absolute effects^*^ (95% CI)		**Relative effect (95% CI)**	**№ of participants (studies)**	**Certainty of the evidence (GRADE)**	**Comments**
	**Risk with placebo**	**Risk with infliximab**				
**Clinical remission** defined as absence of any draining fistulas at ≥ 2 consecutive visits, at a median 12 weeks	129 per 1000	460 per 1000 (178 to 1000)	**RR 3.57** (1.38 to 9.25)	94 (1 study)	⊕⊝⊝⊝ **Very low**^a^	—
**Clinical response** defined as reduction of 50% in the number of draining fistulas at ≥ 2 consecutive visits, at a median 12 weeks	200 per 1000	388 per 1000 (220 to 682)	**RR 1.94** (1.10 to 3.41)	181 (2 studies)	⊕⊝⊝⊝ **Very low**^a^	—
**Withdrawals due to adverse events**	3 per 1000	7.5 per 1000 (0 to 152)	**RR 2.50** (0.12 to 50.54)	94 (1 study)	⊕⊝⊝⊝ **Very low**^a^	—
*The risk in the intervention group (and its 95% confidence interval) is based on the assumed risk in the comparison group and the **relative effect** of the intervention (and its 95% CI). **CI:** confidence interval; **RR:** risk ratio.						
**GRADE Working Group grades of evidence** **High certainty:** we are very confident that the true effect lies close to that of the estimate of the effect. **Moderate certainty:** we are moderately confident in the effect estimate: the true effect is likely to be close to the estimate of the effect, but there is a possibility that it is substantially different. **Low certainty:** our confidence in the effect estimate is limited: the true effect may be substantially different from the estimate of the effect. **Very low certainty:** we have very little confidence in the effect estimate: the true effect is likely to be substantially different from the estimate of effect.						

^a^ Downgraded two levels due to very serious concerns with risk of bias for randomisation, blinding and selective reporting, and one level due to serious concerns with imprecision due to low event numbers.

**Summary of findings 3:** Infliximab 5 mg/kg and purine analogues compared to purine analogues alone

**Infliximab and purine analogues compared to purine analogues alone**						
**Patient or population:** active Crohn's disease **Setting:** hospitals and tertiary centres (Austria, Belgium, Canada, China, Denmark, France, Germany, Greece, Israel, Mexico, the Netherlands, Norway, Spain, Sweden, UK, USA) **Intervention:** infliximab and purine analogues **Comparison:** purine analogues						
**Outcomes**	Anticipated absolute effects^*^ (95% CI)		**Relative effect (95% CI)**	**№ of participants (studies)**	**Certainty of the evidence (GRADE)**	**Comments**
	**Risk with purine analogues**	**Risk with infliximab and purine analogues**				
**Clinical remission** as defined by the studies at weeks 24–26	314 per 1000	604 per 1000 (499 to 728)	**RR 1.92** (1.59 to 2.32)	630 (4 studies)	⊕⊕⊕⊝ **Moderate**^a^	—
**Clinical response** as defined by the studies at week 26	371 per 1000	608 per 1000 (486 to 760)	**RR 1.64** (1.31 to 2.05)	355 (2 studies)	⊕⊕⊕⊝ **Moderate**^a^	—
**Withdrawals due to adverse events** at week 26	205 per 1000	179 per 1000 (129 to 248)	**RR 0.87** (0.63 to 1.21)	603 (4 studies)	⊕⊕⊝⊝ **Low**^b^	—
*The risk in the intervention group (and its 95% confidence interval) is based on the assumed risk in the comparison group and the **relative effect** of the intervention (and its 95% CI). **CI:** confidence interval; **RR:** risk ratio.						
**GRADE Working Group grades of evidence** **High certainty:** we are very confident that the true effect lies close to that of the estimate of the effect. **Moderate certainty:** we are moderately confident in the effect estimate: the true effect is likely to be close to the estimate of the effect, but there is a possibility that it is substantially different. **Low certainty:** our confidence in the effect estimate is limited: the true effect may be substantially different from the estimate of the effect. **Very low certainty:** we have very little confidence in the effect estimate: the true effect is likely to be substantially different from the estimate of effect.						

^a^ Downgraded one level due to serious concerns with risk of bias for randomisation, blinding and selective reporting. ^b^ Downgraded one level due to serious concerns with risk of bias for randomisation, blinding and selective reporting, and one level due to serious concerns with imprecision from low event numbers.

**Summary of findings 4:** Infliximab 5 mg/kg compared to purine analogues

**Infliximab compared to purine analogues**						
**Patient or population:** active Crohn's disease **Setting:** hospitals and tertiary centres (Belgium, Canada, China, Denmark, Sweden, USA) **Intervention:** infliximab **Comparison:** purine analogues						
**Outcomes**	Anticipated absolute effects^*^ (95% CI)		**Relative effect (95% CI)**	**№ of participants (studies)**	**Certainty of the evidence (GRADE)**	**Comments**
	**Risk with purine analogues**	**Risk with infliximab**				
**Clinical remission** at week 26	320 per 1000	480 per 1000 (368 to 624)	**RR 1.50** (1.15 to 1.95)	355 (2 studies)	⊕⊕⊝⊝ **Low**^a^	—
**Clinical response** at week 26	382 per 1000	550 per 1000 (432 to 695)	**RR 1.44** (1.13 to 1.82)	355 (2 studies)	⊕⊕⊝⊝ **Low**^a^	—
**Withdrawals due to adverse events**	241 per 1000	169 per 1000 (111 to 255)	**RR 0.70** (0.46 to 1.06)	355 (2 studies)	⊕⊕⊝⊝ **Low**^a^	—
*The risk in the intervention group (and its 95% confidence interval) is based on the assumed risk in the comparison group and the **relative effect** of the intervention (and its 95% CI). **CI:** confidence interval; **RR:** risk ratio.						
**GRADE Working Group grades of evidence** **High certainty:** we are very confident that the true effect lies close to that of the estimate of the effect. **Moderate certainty:** we are moderately confident in the effect estimate: the true effect is likely to be close to the estimate of the effect, but there is a possibility that it is substantially different. **Low certainty:** our confidence in the effect estimate is limited: the true effect may be substantially different from the estimate of the effect. **Very low certainty:** we have very little confidence in the effect estimate: the true effect is likely to be substantially different from the estimate of effect.						

^a^ Downgraded one level due to serious concerns with risk of bias for randomisation, blinding and selective reporting, and one level due to serious concerns with imprecision from low event numbers.

**Summary of findings 5:** Infliximab 5 mg/kg compared to infliximab 10 mg/kg

**Infliximab 5 mg/kg compared to infliximab 10 mg/kg**						
**Patient or population:** active Crohn's disease **Setting:** hospitals and tertiary centres (Belgium, England, Germany, the Netherlands, USA) **Intervention:** infliximab 5 mg/kg **Comparison:** infliximab 10 mg/kg						
**Outcomes**	Anticipated absolute effects^*^ (95% CI)		**Relative effect (95% CI)**	**№ of participants (studies)**	**Certainty of the evidence (GRADE)**	**Comments**
	**Risk with infliximab 10 mg/kg**	**Risk with infliximab 5 mg/kg**				
**Clinical remission** defined as CDAI < 150 at week 4	393 per 1000	703 per 1000 (417 to 1000)	**RR 1.79** (1.06 to 3.02)	55 (1 study)	⊕⊝⊝⊝ **Very low**^a^	—
**Clinical response** defined as reduction in CDAI by 70 points at week 4	500 per 1000	815 per 1000 (540 to 1000)	**RR 1.63** (1.08 to 2.46)	55 (1 study)	⊕⊝⊝⊝ **Very low**^a^	—
**Withdrawals due to adverse events**	—	—	—	—	—	—
*The risk in the intervention group (and its 95% confidence interval) is based on the assumed risk in the comparison group and the **relative effect** of the intervention (and its 95% CI). **CDAI:** Crohn's Disease Activity Index; **CI:** confidence interval; **RR:** risk ratio.						
**GRADE Working Group grades of evidence** **High certainty:** we are very confident that the true effect lies close to that of the estimate of the effect. **Moderate certainty:** we are moderately confident in the effect estimate: the true effect is likely to be close to the estimate of the effect, but there is a possibility that it is substantially different. **Low certainty:** our confidence in the effect estimate is limited: the true effect may be substantially different from the estimate of the effect. **Very low certainty:** we have very little confidence in the effect estimate: the true effect is likely to be substantially different from the estimate of effect.						

^a^ Downgraded one level due to serious concerns with risk of bias (selective reporting and unclear randomisation), and two levels due to concerns with imprecision due to low event numbers.

**Summary of findings 6:** Infliximab 5 mg/kg compared to infliximab 10 mg/kg for exclusively fistulating population

**Infliximab 5 mg/kg compared to infliximab 10 mg/kg for exclusively fistulating population**						
**Patient or population:** active Crohn's disease **Setting:** not reported (multiple countries) **Intervention:** infliximab 5 mg/kg **Comparison:** infliximab 10 mg/kg						
**Outcomes**	Anticipated absolute effects^*^ (95% CI)		**Relative effect (95% CI)**	**№ of participants (studies)**	**Certainty of the evidence (GRADE)**	**Comments**
	**Risk with infliximab 10 mg/kg**	**Risk with infliximab 5 mg/kg**				
**Clinical remission** defined as absence of any draining fistulas at ≥ 2 consecutive visits, at a median length of time of 12 weeks	375 per 1000	548 per 1000 (315 to 949)	**RR 1.46** (0.84 to 2.53)	63 (1 study)	⊕⊝⊝⊝ **Very low**^a^	—
**Clinical response** defined as reduction of 50% in the number of draining fistula at ≥ 2 consecutive visits, at a median length of time of 12 weeks	563 per 1000	675 per 1000 (462 to 1000)	**RR 1.20** (0.82 to 1.78)	63 (1 study)	⊕⊝⊝⊝ **Very low**^a^	—
**Withdrawals due to adverse events**	31 per 1000	32 per 1000 (2 to 505)	**RR 1.03** (0.07 to 15.79)	63 (1 study)	⊕⊝⊝⊝ **Very low**^a^	—
*The risk in the intervention group (and its 95% confidence interval) is based on the assumed risk in the comparison group and the **relative effect** of the intervention (and its 95% CI). **CI:** confidence interval; **RR:** risk ratio.						
**GRADE Working Group grades of evidence** **High certainty:** we are very confident that the true effect lies close to that of the estimate of the effect. **Moderate certainty:** we are moderately confident in the effect estimate: the true effect is likely to be close to the estimate of the effect, but there is a possibility that it is substantially different. **Low certainty:** our confidence in the effect estimate is limited: the true effect may be substantially different from the estimate of the effect. **Very low certainty:** we have very little confidence in the effect estimate: the true effect is likely to be substantially different from the estimate of effect.						

^a^ Downgraded one level due to serious concerns with risk of bias for randomisation, blinding and selective reporting, and one level due to serious concerns with imprecision due to low event numbers.

**Summary of findings 7:** Infliximab 5 mg/kg compared to infliximab 20 mg/kg

**Infliximab 5 mg/kg compared to infliximab 20 mg/kg**						
**Patient or population:** active Crohn's disease **Setting:** hospitals and tertiary centres (Belgium, England, Germany, the Netherlands, USA) **Intervention:** infliximab 5 mg/kg **Comparison:** infliximab 20 mg/kg						
**Outcomes**	Anticipated absolute effects^*^ (95% CI)		**Relative effect** **(95% CI)**	**№ of participants** **(studies)**	**Certainty of the evidence** **(GRADE)**	**Comments**
	**Risk with infliximab 20 mg/kg**	**Risk with infliximab 5 mg/kg**				
**Clinical remission** at week 4	393 per 1000	703 per 1000 (417 to 1000)	**RR 1.79** (1.06 to 3.02)	55 (1 study)	⊕⊝⊝⊝ **Very low**^a^	—
**Clinical response** as defined by the studies week 4	642 per 1000	816 per 1000 (540 to 1000)	**RR 1.27** (0.91 to 1.76)	55 (1 study)	⊕⊝⊝⊝ **Very low**^a^	—
**Withdrawals due to adverse events**	—		—	—	—	—
*The risk in the intervention group (and its 95% confidence interval) is based on the assumed risk in the comparison group and the **relative effect** of the intervention (and its 95% CI). **CI:** confidence interval; **RR:** risk ratio.						
**GRADE Working Group grades of evidence** **High certainty:** we are very confident that the true effect lies close to that of the estimate of the effect. **Moderate certainty:** we are moderately confident in the effect estimate: the true effect is likely to be close to the estimate of the effect, but there is a possibility that it is substantially different. **Low certainty:** our confidence in the effect estimate is limited: the true effect may be substantially different from the estimate of the effect. **Very low certainty:** we have very little confidence in the effect estimate: the true effect is likely to be substantially different from the estimate of effect.						

^a^ Downgraded one level due to serious concerns with risk of bias (selective reporting and unclear randomisation), and two levels due to very serious concerns with imprecision due to very low event numbers.

**Summary of findings 8:** Infliximab 10 mg/kg compared to infliximab 20 mg/kg

**Infliximab 10 mg/kg compared to infliximab 20 mg/kg**						
**Patient or population:** active Crohn's disease **Setting:** hospitals and tertiary centres (Belgium, England, Germany, the Netherlands, USA) **Intervention:** infliximab 10 mg/kg **Comparison:** infliximab 20 mg/kg						
**Outcomes**	Anticipated absolute effects^*^ (95% CI)		**Relative effect (95% CI)**	**№ of participants (studies)**	**Certainty of the evidence (GRADE)**	**Comments**
	**Risk with infliximab 20 mg/kg**	**Risk with infliximab 10 mg/kg**				
**Clinical remission** defined as CDAI < 150 at week 4	393 per 1000	393 per 1000 (204 to 755)	**RR 1.00** (0.52 to 1.92)	56 (1 study)	⊕⊝⊝⊝ **Very low**^a^	—
**Clinical response** defined by reduction of CDAI score by ≥ 70 at week 4	643 per 1000	501 per 1000 (315 to 791)	**RR 0.78** (0.49 to 1.23)	56 (1 study)	⊕⊝⊝⊝ **Very low**^a^	—
**Withdrawals due to adverse events**	—		—	—	—	—
*The risk in the intervention group (and its 95% confidence interval) is based on the assumed risk in the comparison group and the **relative effect** of the intervention (and its 95% CI). **CDAI:** Crohn's Disease Activity Index; **CI:** confidence interval; **RR:** risk ratio.						
**GRADE Working Group grades of evidence** **High certainty:** we are very confident that the true effect lies close to that of the estimate of the effect. **Moderate certainty:** we are moderately confident in the effect estimate: the true effect is likely to be close to the estimate of the effect, but there is a possibility that it is substantially different. **Low certainty:** our confidence in the effect estimate is limited: the true effect may be substantially different from the estimate of the effect. **Very low certainty:** we have very little confidence in the effect estimate: the true effect is likely to be substantially different from the estimate of effect.						

^a^ Downgraded one level due to serious concerns with risk of bias (selective reporting and unclear randomisation), and two levels due to very serious concerns with imprecision due to very low event numbers.

**Summary of findings 9:** Infliximab 5 mg/kg compared to CT‐P13 biosimilar 5 mg/kg

**Infliximab compared to biosimilar**						
**Patient or population:** active Crohn's disease **Setting:** 58 centres in 16 countries (Belgium, Brazil, Denmark, France, Germany, Hungary, Italy, Israel, Mexico, the Netherlands, Poland, Republic of Korea, Romania, Russia, Ukraine, USA) **Intervention:** infliximab 5 mg/kg **Comparison:** CT‐P13 biosimilar 5 mg/kg						
**Outcomes**	Anticipated absolute effects^*^ (95% CI)		**Relative effect (95% CI)**	**№ of participants (studies)**	**Certainty of the evidence (GRADE)**	**Comments**
	**Risk with infliximab**	Risk with CT‐P13 biosimilar				
**Clinical remission** defined as CDAI < 150 at week 6	441 per 1000	432 per 1000 (317 to 582)	**RR 0.98** (0.72 to 1.32)	220 (1 study)	⊕⊕⊝⊝ **Low**^a^	—
**Clinical response** defined by reduction of CDAI score by ≥ 100 at week 6	631 per 1000	612 per 1000 (498 to 757)	**RR 0.97** (0.79 to 1.20)	220 (1 study)	⊕⊕⊝⊝ **Low**^a^	—
**Withdrawals due to adverse events**	153 per 1000	193 per 1000 (107 to 344)	**RR 1.26** (0.70 to 2.25)	220 (1 study)	⊕⊕⊝⊝ **Low**^a^	—
*The risk in the intervention group (and its 95% confidence interval) is based on the assumed risk in the comparison group and the **relative effect** of the intervention (and its 95% CI). **CDAI:** Crohn's Disease Activity Index; **CI:** confidence interval; **RR:** risk ratio.						
**GRADE Working Group grades of evidence** **High certainty:** we are very confident that the true effect lies close to that of the estimate of the effect. **Moderate certainty:** we are moderately confident in the effect estimate: the true effect is likely to be close to the estimate of the effect, but there is a possibility that it is substantially different. **Low certainty:** our confidence in the effect estimate is limited: the true effect may be substantially different from the estimate of the effect. **Very low certainty:** we have very little confidence in the effect estimate: the true effect is likely to be substantially different from the estimate of effect.						

^a^ Downgraded two levels due to very serious concerns with imprecision due to very low event numbers.

## Background

### Description of the condition

Crohn's disease (CD) is a chronic idiopathic disease characterised by transmural inflammation of the gastrointestinal tract (Neurath 2012). Although symptoms can vary, people with CD commonly present with abdominal pain, diarrhoea and weight loss. CD is not limited to the intestines. Between 25% and 70% of people with CD experience extraintestinal manifestations such as arthritis, osteoporosis, uveitis, erythema nodosum, psoriasis, ankylosing spondylitis, sacroiliitis, oral aphthous stomatitis, pyoderma gangrenosum and primary sclerosing cholangitis (Peyrin‐Biroulet 2017). While CD may present at any age, the peak ages of diagnosis are in the second and third decades of life (Vind 2006). Fistulising Crohn's is a more severe form of the disease.

In North America, the annual incidence of CD ranges from 3.1 to 14.6 cases per 100,000 person‐years, with a prevalence between 26 and 199 cases per 100,000 people (Loftus 2004). The most recent estimates of the prevalence of inflammatory bowel disease (IBD) in the UK are 9 to 144/100,000 for CD (Jones 2019).

Although the precise aetiology of CD remains unknown, it is believed that a genetic predisposition combined with exogenous (intestinal flora) and endogenous (epithelial cell function and immune cell function) factors contribute to the development of inflammation in the intestinal mucosa (Baumgart 2007). This dysregulated inflammatory response creates an imbalance between pro‐inflammatory and anti‐inflammatory mediators. Available CD therapies attempt to attenuate the resulting inflammatory response (Lichtenstein 2006).

Since CD is characterised by alternating states of active and quiescent disease, the therapeutic goal is to induce and maintain the remission of symptoms, as well as endoscopically and radiologically. Treatment guidelines recommend a sequential step‐up approach that focusses on treating the acute disease (i.e. inducing clinical remission) and maintaining response (Lamb 2019). At the bottom of the pyramid are treatments such as elimination diets, antibiotics and glucocorticoids, which may be less effective but are associated with limited systemic toxicity. In a step‐up manner, non‐responders are subsequently treated with more aggressive, potentially toxic medications including immunosuppressives (i.e. azathioprine, 6‐mercaptopurine and methotrexate) as well as biological drugs (i.e. infliximab, adalimumab, certolizumab, natalizumab, ustekinumab and vedolizumab) in an attempt to induce and maintain remission (D'Haens 2008; Ha 2014; Okabayashi 2022; Rawla 2018).

Conventional treatment options for people with active CD include systemic corticosteroids (e.g. hydrocortisone, prednisolone), locally acting corticosteroids (e.g. budesonide), and immunosuppressives (e.g. azathioprine, 6‐mercaptopurine and methotrexate). However, corticosteroid resistance develops in 16% to 20% of people with active CD (Faubion 2001; Munkholm 1995), and many people do not respond to immunosuppressives.

### Description of the intervention

Infliximab is a human–mouse chimeric monoclonal antibody that works by binding and neutralising the pro‐inflammatory cytokine tumour necrosis factor‐alpha (TNF‐α), which is found in high levels in the blood serum, mucosa and stool of people with CD. Infliximab is able to render this cytokine biologically inactive (Knight 1993).

Infliximab belongs in a category of drugs called biologicals. Biologicals are produced, either entirely or partly, from biological sources, and target the immune system's inflammatory responses.

### How the intervention might work

Cytokines, which are intercellular mediators, control the inflammatory process in CD (Sartor 1994). Chronic local inflammation occurs as a result of mucosal overproduction of pro‐inflammatory cytokines (Rogler 1998). One of several pro‐inflammatory mediators, TNF‐α, plays a key role in numerous inflammatory processes including CD, rheumatoid arthritis and other granulomatous diseases (Bell 2000). Drugs that inhibit the actions of TNF‐α, including infliximab, are termed TNF‐α antagonists.

In people with CD, the local effects of infliximab on inflamed bowel mucosa lead to a reduction in TNF‐α‐expressing cells within four weeks of starting treatment. Compared to people treated with placebo, the colonic lamina propria of people treated with infliximab have demonstrated greater than 50% reduction of cells that produce TNF‐α including CD4+, CD8+, CD68+ monocytes and macrophages (Baert 1999). Treatment with infliximab also leads to a reduction in the number of interferon‐gamma‐ and TNF‐α‐producing mononuclear cells in the lamina propria of people with CD (Plevy 1997). Infliximab reduces the expression of adhesion molecules (ICAM‐1 (intercellular adhesion molecule‐1) and LFA‐1 (lymphocyte function‐associated antigen‐1)) (Baert 1999). Along with histological repair, infliximab appears to be effective for achieving endoscopic healing, which is correlated with a reduction in disease activity as shown by D'Haens 1999.

### Why it is important to do this review

The discovery of infliximab was a turning point in the management of CD. Although biologicals can provide rapid and effective clinical response, mucosal healing, improved quality of life and reduced need for surgery, whether these drugs are a cost‐effective choice is debated (Cote‐Daigenault 2015).

Infliximab is a promising therapeutic option for people with moderate‐to‐severe active CD who fail to respond to conventional therapy or have fistulising disease. The ACCENT‐I and ACCENT‐II trials suggest that infliximab is effective for inducing and maintaining remission (Hanauer 2002; Sands 2004a). ACCENT‐I demonstrated that scheduled infliximab treatment may be more effective than sporadic treatment, and it can lead to a greater probability of mucosal healing and decreased hospital admissions (Baert 2010).

Although a previous Cochrane Review assessed the efficacy and safety of TNF‐α antagonists for induction of remission in CD (Akobeng 2003), this review will focus on infliximab. It will provide an up‐to‐date summary of the benefits and harms of infliximab used for the treatment of moderate‐to‐severe CD.

## Objectives

To evaluate the benefits and harms of infliximab alone or in combination with another agent for induction of remission in Crohn's disease compared to placebo or active medical therapies.

## Methods

### Criteria for considering studies for this review

#### Types of studies

We considered all types of randomised controlled trials (RCT) for inclusion. Quasi‐randomised trials (using inappropriate randomisation methods) were ineligible.

#### Types of participants

We considered adults aged greater than 18 years with active CD as defined by the study authors (as per conventional clinical, radiological or endoscopic criteria) for inclusion. Participants with all disease locations and behaviours as defined by the primary study. There were no restrictions applied for sex, disease duration or previous medication exposure.

We included studies with CD as a subset of a wider IBD population only if they offered separate data for the participants with CD. We included studies reporting on CD subsets (e.g. fistulating population), and analysed them separately from the general CD populations.

#### Types of interventions

We included studies analysing infliximab, alone or in combination with another agent, compared to placebo or active medical therapies for induction of remission in people with CD.

In studies where purine analogue use exceeded 50% amongst all participants, we considered the purine analogues as part of the intervention.

We excluded surgical interventions from this review.

#### Types of outcome measures

We included both dichotomous and continuous outcomes.

##### Primary outcomes

- **Clinical remission** (as defined by the included studies), as measured at the primary endpoint of the study, but not later than 26 weeks
- **Clinical response** (as defined by the included studies), as measured at the primary endpoint of the study, but not later than 26 weeks
- **Withdrawals due to adverse events** for the duration of the follow‐up

##### Secondary outcomes

- **Endoscopic remission** (as defined by the included studies), as measured at the primary endpoint of the study, but not later than 26 weeks
- **Histological remission** (as defined by the included studies), as measured at the primary endpoint of the study, but not later than 26 weeks
- **Endoscopic response** (as defined by the included studies), as measured at the primary endpoint of the study, but not later than 26 weeks
- **Serious adverse events** for the duration of the follow‐up of the included studies
- **Total adverse events** for the duration of the follow‐up of the included studies

### Search methods for identification of studies

#### Electronic searches

On 31 August 2021 and 4 March 2023, the Cochrane Gut Information Specialist searched the following sources.

- Cochrane Central Register of Controlled Trials (CENTRAL) via the Cochrane Library (Issue 2, 2023)
- MEDLINE via OvidSP (1946 to 2 March 2023)
- Embase via OvidSP (1974 to week 8 2023)
- ClinicalTrials.gov (clinicaltrials.gov/; searched 4 March 2023)
- World Health Organization International Clinical Trials Registry Platform (WHO ICTRP; trialsearch.who.int/; searched 4 March 2023)

We did not apply any date, language, document type or publication status limitations to this search. For the search strategies, see Appendix 1.

#### Searching other resources

We searched the references of included studies and applicable systematic reviews to identify additional studies. We searched conference proceedings from Digestive Disease Week, the European Crohn's and Colitis Organisation Congress and United European Gastroenterology Week to identify studies reported in abstract form only for the last 24 months to cover for possible indexing delays between the publication of conference abstracts and their indexing in Embase.

We also corresponded with authors and experts in the field to identify unpublished data.

### Data collection and analysis

#### Selection of studies

Four review authors (GWM, SJR, ME, AMD) worked in pairs to assess publications identified by the search strategy to determine eligibility based on the above inclusion criteria. We resolved any disagreements by discussion and consensus amongst the review authors. If consensus could not be reached, we consulted a fifth review author (MG).

#### Data extraction and management

We collected information from included studies using a standardised data collection form. Pairs of review authors independently extracted data. We resolved disagreements by discussion and consensus. If consensus could not be reached, we consulted a fifth review author (MG).

The extracted data included the following.

- General information (title, journal, year, publication type)
- Study information (design, methods of randomisation, concealment of allocation and blinding, power calculation, a priori and post hoc analyses)
- Intervention and control (type and dose of medication; placebo or active comparator)
- Eligibility (total number of participants screened and randomised)
- Baseline characteristics for each arm (age, sex, ethnicity, disease severity, concurrent medications, prior medications)
- Follow‐up (length of follow‐up, assessment of treatment compliance, withdrawals, number of participants lost to follow‐up)
- Outcomes (primary and secondary outcomes)

#### Assessment of risk of bias in included studies

Four review authors (GWM, SJR, ME, AMD) worked in pairs to assess the risk of bias of each included study using the Cochrane RoB 1 tool (Higgins 2011). We assessed the following factors.

- Random sequence generation (i.e. randomisation method)
- Allocation concealment (selection bias)
- Blinding of participants and personnel (performance bias)
- Blinding of outcome assessment (detection bias)
- Incomplete outcome data (i.e. methods used by investigators to deal with attrition)
- Selective reporting (i.e. investigators reported all outcomes)
- Other bias (i.e. any other factor that could have increased bias)

We judged studies at high, low or unclear risk of bias. We resolved disagreements by consensus via discussion. If consensus could not be reached, we consulted a fifth review author (MG).

#### Measures of treatment effect

We analysed all data on an intention‐to‐treat (ITT) basis using Review Manager Web (RevMan Web 2020).

For dichotomous outcomes, we expressed the treatment effect as risk ratios (RR) with corresponding 95% confidence intervals (CI). For continuous outcomes, we expressed the treatment effect as mean differences (MD) with 95% CIs when studies used the same scale. When studies used different scales to measure the same underlying construct, we calculated the standardised mean difference (SMD) and 95% CI.

As the included studies differed in their chosen primary outcome data time points, we included different time points in our meta‐analyses, which we then investigated for heterogeneity (Assessment of heterogeneity). We extracted no further time‐to‐event data.

#### Unit of analysis issues

The participant was the unit of analysis. For studies comparing more than two intervention groups, we made multiple pairwise comparisons between all possible pairs of intervention groups. To avoid double counting, we divided shared intervention groups evenly amongst the comparisons. For dichotomous outcomes, we divided both the number of events and the total number of participants. For continuous outcomes, we only divided the total number of participants, and left the means and standard deviations unchanged.

We included cross‐over studies, but only pooled their data if they were reported separately before and after cross‐over, and we only used pre‐cross‐over data.

#### Dealing with missing data

We based our analysis on the data made available by the study authors. We contacted study authors to request missing data, data that were not reported in sufficient detail or unclear data.

For efficacy outcomes, we used the numbers randomised as denominators. For numerators, we used the numbers as reported by the study authors. Participants with missing or unclear data were assumed to be treatment failures.

For safety outcomes, we considered participants with missing or unclear withdrawal data as withdrawals due to adverse events. The denominators used for this outcome were as reported by the study authors. For serious and total adverse events, we used the numbers of events per participant, as reported by the study authors. We discarded outcome data reported for mixes of randomised and non‐randomised participants.

We employed the same methods in our sensitivity analyses.

#### Assessment of heterogeneity

We scrutinised studies to ensure that they were clinically homogeneous in terms of participants, interventions, comparators and outcomes. To test for statistical heterogeneity, we used a Chi² test. A P value of less than 0.1 gave an indication of the presence of heterogeneity. We quantified inconsistency using the I² statistic. We interpreted the thresholds as follows (Higgins 2022).

- 0% to 40%: might not be important
- 30% to 60%: may represent moderate heterogeneity
- 50% to 90%; may represent substantial heterogeneity
- 75% to 100%: considerable heterogeneity

We examined possible explanations for heterogeneity when there were sufficient data available, including factors such as participant characteristics (e.g. age, sex), condition severity, healthcare system and country. We did not pool data in a meta‐analysis if there was a considerable degree of statistical heterogeneity (I² greater than 75%). In the case of considerable statistical heterogeneity, we investigated whether this could be explained on clinical grounds or risk of bias, in which case, we aimed to conduct sensitivity analyses. If we found no reasons for the considerable statistical heterogeneity, we presented the results narratively, in detail.

#### Assessment of reporting biases

Our use of an inclusive search strategy minimised most reporting biases. We aimed to investigate publication bias using a funnel plot for outcomes with 10 or more studies and determine the magnitude of publication bias by visual inspection of the asymmetry of the funnel plot or other methods mentioned in the *Cochrane Handbook for Systematic Reviews of Interventions* (Higgins 2022). We would also have tested funnel plot asymmetry by performing a linear regression of the intervention effect estimate against its standard error, weighted by the inverse of the variance of the intervention effect estimate (Egger 1997).

#### Data synthesis

We combined data from individual trials for meta‐analysis when the interventions, participant groups and outcomes were sufficiently similar (as determined by consensus). For dichotomous outcomes, we calculated the pooled RR and 95% CI. For continuous outcomes, we calculated the pooled MD and corresponding 95% CI when studies used the same scale. When studies used different scales to measure the same underlying construct, we calculated the SMD and 95% CI. We used a random‐effects model to pool data. If there was a high degree of heterogeneity (I^2^ of 75 or greater), we did not pool data for meta‐analysis.

We reported data that could not be meta‐analysed in narrative form using the SWiM guidance.

#### Subgroup analysis and investigation of heterogeneity

Planned subgroup analyses were:

- different drug doses and dosing frequencies;
- concomitant immunosuppressant medication use and
- different disease behaviours.

If heterogeneity was detected, we investigated possible causes, and addressed them using methods described in Higgins 2022.

#### Sensitivity analysis

Planned sensitivity analyses to examine the impact of the following variables on the pooled effect were:

- random‐effects versus fixed‐effect modelling;
- low risk of bias versus unclear or high risk of bias;
- relevant loss to follow‐up (greater than 10%): best‐case versus worst‐case scenario;
- full‐text manuscript versus abstract or unpublished studies.

#### Summary of findings and assessment of the certainty of the evidence

We presented a summary of our findings and GRADE decisions for all comparisons for all three of our primary outcomes in the summary of findings tables.

We assessed the overall certainty of the evidence for the primary and secondary outcomes using the GRADE approach (Guyatt 2008; Schünemann 2011). Evidence retrieved from RCTs is usually regarded as high certainty. However, the certainty rating may be downgraded as a result of:

- risk of bias;
- indirect evidence;
- inconsistency (unexplained heterogeneity);
- imprecision and
- publication bias.

##### GRADE Working Group grades of evidence

- High certainty: we are very confident that the true effect lies close to that of the estimate of the effect.
- Moderate certainty: we are moderately confident in the effect estimate; the true effect is likely to be close to the estimate of the effect, but there is a possibility that it is substantially different.
- Low certainty: our confidence in the effect estimate is limited; the true effect may be substantially different from the estimate of the effect.
- Very low certainty: we have very little confidence in the effect estimate; the true effect is likely to be substantially different from the estimate of effect.

## Results

### Description of studies

#### Results of the search

Our search, conducted up to March 2023, identified 7066 records. After removing duplicates, 7055 records underwent title and abstract screening to assess eligibility, of which we excluded 6982 records. The remaining 73 records underwent full‐text review, of which we excluded 48 records (47 studies) with reasons, and found one ongoing study (one record).

We included 10 RCTs with 1101 randomised participants.

The results of the search are presented in the study flow diagram (Figure 1).

**1:**
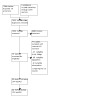
Study flow diagram.

#### Included studies

A summary of key characteristics and interventions across the included studies is shown in Table 10 and Table 11. The outcome data can be found in Table 12. See also the Characteristics of included studies table.

**1:** Included studies' characteristics

**Study ID**	**Group interventions**	**Numbers randomised**	**Purine analogues use**	**Biological naive or not**
Colombel 2010	**IG1:** IFX 5 mg/kg **IG2:** combination therapy **CG:** AZA 2.5 mg/kg	Total randomised: 508 **IG1:** 169 **IG2:** 169 **CG:** 170	Part of the intervention	Naive
D'Haens 1999	**IG1:** IFX 5 mg **IG2:** IFX 10 mg **IG3:** IFX 20 mg **CG:** placebo 0.1% human serum albumin	Total randomised: 30 **IG1:** 7 **IG2:** 7 **IG3:** 8 **CG:** 8	Concomitant azathioprine **IG1:** 3/7 **IG2:** 1/7 **IG3:** 5/8 **CG:** 3/8	Naive
D'Haens 2008	**IG:** IFX 5 mg/kg + AZA 2–2.5 mg/kg **CG:** corticosteroids	Total randomised: 133 **IG1:** 67 **CG:** 66	Part of the intervention	Naive
Duan 2013	**IG1:** IFX 5 mg/kg **IG2:** AZA 2.5 mg/kg + IFX 5 mg/kg **CG:** AZA 2.5 mg/kg	Total randomised: 24 **IG1:** 8 **IG2:** 8 **CG:** 8	Part of the intervention	Unclear
Hanauer 2002	**IG1:** IFX infusion, regimen 1 **IG2:** IFX infusion, regimen 2 **CG:** placebo	Total randomised: 238 **IG1:** 79 **IG2:** 81 **CG:** 78	Only reported for the whole cohort (238 participants) 5‐ASA: 288 AZA/6‐MP: 63 Methotrexate: 13	Not naive
Lemann 2006	**IG:** IFX 5 mg/kg **CG:** placebo	Total randomised: 115 **IG:** 57 **CG:** 58	Part of the intervention	Naive
Present 1999	**IG1:** IFX 5 mg/kg **IG2:** IFX 10 mg/kg **CG:** placebo	Total randomised: 94 **IG1:** 31 **IG2:** 32 **CG:** 31	Mercaptopurine or azathioprine: **IG1:** 12/31 **IG2:** 17/32 **CG:** 9/31	Naive
Sands 2004b	**IG:** IFX 5 mg/kg **CG:** placebo	Total randomised: 87 **IG:** 43 **CG:** 44	Mercaptopurine or azathioprine: Overall: 53/87 (61%)	Not naive
Targan 1997	**IG1:** cA2 at 5 mg/kg **IG2:** cA2 at 10 mg/kg **IG3:** cA2 at 20 mg/kg **CG:** 0.1% human serum albumin	Total randomised: 108 **IG1:** 27 **IG2:** 28 **IG3:** 28 **CG:** 25	Mercaptopurine: **IG1:** 4 **IG2:** 4 **IG3:** 4 **CG:** 4 Azathioprine: **IG1:** 5 **IG2:** 4 **IG3:** 8 **CG:** 7	Naive
Ye 2019	**IG:** CT‐P13 biosimilar 5 mg/kg **CG:** IFX 5 mg/kg	Total number randomised: 220 **IG:** 111 **CG:** 109	Mercaptopurine or azathioprine: **IG:** 84/111 **CG:** 80/109	Naive

5‐ASA: 5‐aminosalicylic acid; 6‐MP: 6‐mercaptopurine; AZA: azathioprine; cA2: early name for infliximab; IFX: infliximab; CG: control group; IG: intervention group.

**2:** Included studies' intervention details

**Study ID**	**Intervention medications**	**Previous experience of biological therapy**	**Medications up to starting study**	**Medications that had to be discontinued prior to starting study**	**Mandatory medications per study protocol**	**Concomitant medications during study**
Colombel 2010	**IG1:** IFX only. IV infusion of IFX 5 mg/kg at weeks 0, 2, 6, 14 and 22, plus daily oral placebo capsules through 30 weeks **IG2:** combination therapy. IV infusion of IFX 5 mg/kg at weeks 0, 2, 6, 14 and 22 plus daily oral AZA capsules 2.5 mg/kg through 30 weeks **CG:** AZA only. Daily oral capsules 2.5 mg/kg through 30 weeks plus placebo IV infusion at weeks 0, 2, 6, 14 and 22	None All participants naive	**IG1** No steroids: 117 Steroids < 20 mg: 19 Steroids > 20 mg: 33 Budesonide: 28 5‐ASA: 87 **IG2** No steroids: 122 Steroids < 20 mg: 14 Steroids > 20 mg: 33 Budesonide: 19 5‐ASA: 85 **CG** No steroids: 130 Steroids < 20 mg: 14 Steroids > 20 mg: 26 Budesonide: 25 5‐ASA: 104	NR	NR	Systemic steroids **IG1:** 60 **IG2:** 58 **CG:** 60
D'Haens 1999	**IG1:** single IV dose IFX 5 mg/kg **IG2:** single IV dose IFX 10 mg/kg **IG3:** single IV dose IFX 20 mg/kg **CG:** single IV dose of placebo 0.1% human serum albumin	None *(exclusion criteria)*	Steroids: **IG1:** 4 **IG2:** 3 **IG3:** 4 **CG:** 5 AZA: **IG1:** 4 **IG2:** 3 **IG3:** 4 **CG:** 5	NR	NR	NR
D'Haens 2008	**IG1:** single IV dose IFX 5 mg/kg at weeks 0, 2, 6 with AZA 2–2.5 mg/kg per day. If not tolerating AZA, 6‐MP given initial dose of 25 mg each week for 12 weeks with the dose reduced to 15 mg/week thereafter **IG2:** corticosteroids then AZA	0 (exclusion criteria)	0 (exclusion criteria)	NR	NR	**IG1:** AZA 50/65 (77%) MTX 16/65(25%) **IG2:** AZA 38/64 (60%) MTX 8/64 (13%)
Duan 2013	**IG1:** IV IFX 5 mg/kg at weeks 0, 2 and 6 to induce remission followed by IFX 5 mg/kg every 8 weeks to maintain remission **IG2:** AZA PO 2.5 mg/kg qd, and IV IFX 5 mg/kg at weeks 0, 2 and 6 to induce remission, followed by IFX 5 mg/kg every 8 weeks to maintain remission **CG:** AZA PO 2.5 mg/kg qd	NR	NR	NR	NR	NR
Hanauer 2002	At week 0, all eligible participants received IFX 5 mg/kg IV **IG1:** IFX 5 mg/kg infusions at week 2, 6 and every 8 weeks thereafter until week 46 **IG2:** IFX 10 mg/kg infusions at weeks 2, 6 and every 8 weeks thereafter until week 46 **CG:** placebo infusions at weeks 2, 6 and every 8 weeks thereafter until week 46	Participants were excluded from the study if they had received previous treatment with IFX or any other agent targeted at TNF	5‐ASA: 129 6‐MP and AZA: 63 MTX: 13 Corticosteroids of which: any – 118 > 20 mg/day: 32	Participants not receiving medical therapy had to have discontinued treatment for ≥ 4 weeks before screening	None	Same as medications up to the start of study
Lemann 2006	All participants were treated with AZA/6‐MP 2–3 mg/kg Failure stratum (56 enroled): **IG:** 26 IFX 0, 2 and 6 weeks at 5 mg/kg **CG:** 29 placebo Naive stratum (59 enroled): **IG:** 31 IFX 0, 2 and 6 weeks at 5 mg/kg **CG:** 27 placebo	0 (exclusion criteria)	All participants received steroids: 115 All participants in the failure stratum were on and continued to use AZA: 56/115	5‐ASA, budesonide, artificial nutrition or other immunosuppressive drugs	All participants were treated with AZA 2–3 mg/kg per day or 6‐MP 1.0–1.5 mg/kg per day. Participants previously treated with AZA or 6‐MP (failure stratum) continued their treatment at the same dose; in the naive‐stratum participants were treated with AZA 2.0–2.5 mg/kg per day, started 1 week after the first IFX infusion. The AZA or 6‐MP had to be maintained at a stable dose throughout the study, except for participants who experienced toxicity related to the drug.	NR
Present 1999	**IG1:** IFX 5 mg/kg (0, 2 and 6 weeks) **IG2:** IFX 10 mg/kg (0, 2 and 6 weeks) **CG:** placebo	0	**IG1:** (n = 31) Corticosteroids: 12 6‐MP and AZA: 12 5‐ASA: 17 Antibiotics: 6 **IG2:** (n = 32) Corticosteroids: 10 6‐MP and AZA: 17 5‐ASA: 16 Antibiotics: 11 **CG:** (n = 31) Corticosteroids: 11 6‐MP and AZA: 9 5‐ASA: 19 Antibiotics: 11	Ciclosporin (excluded from the start) IF not already on a stable dose of steroids/AZA/aminosalicylates/MTX medications had to have discontinued ≥ 4 weeks before enrolment	NR	**IG1 + IG2:** Corticosteroids: 21 6‐MP or AZA: 29 5‐ASA: NR Antibiotics: 17 (the numbers were the same as medication up to study, so it seems most of the participants who were on these drugs continued throughout the study, although the paper did not state it) **CG:** Corticosteroids: 11 6‐MP or AZA: 9 5‐ASA: 19 Antibiotics: 11 Study reported IG1 and IG2, concomitant use
Sands 2004b	**IG:** IFX 5 mg/kg, IV, at weeks 14, 22, 30, 38 and 46. Beginning at week 22, participants who had a loss of response were eligible to cross over to maintenance treatment with IFX 10 mg/kg **CG:** placebo (agent, dose and route: NR) at weeks 14, 22, 30, 38 and 46. Beginning at week 22, participants who had a loss of response were eligible to cross over to maintenance treatment with IFX 5 mg/kg	NR	NR	NR	NR	NR
Targan 1997	**IG1:** single dose, IV, cA2 at 5 mg/kg **IG2:** single dose, IV, cA2 at 10 mg/kg **IG3:** single dose, IV, cA2 at 20 mg/kg **CG:** single dose, IV, placebo 0.1% human serum albumin	None *(exclusion criteria)*	n per group (%) Prednisolone (< 20 mg/day PO): **IG1:** 8 **IG2:** 8 **IG3:** 10 **CG:** 10 Prednisolone (≥ 20 mg/day PO): **IG1:** 7 **IG2:** 8 **IG3:** 7 **CG:** 6 6‐MP: **IG1:** 4 **IG2:** 4 **IG3:** 4 **CG:** 4 AZA: **IG1:** 5 **IG2:** 4 **IG3:** 8 **CG:** 7 5‐ASA: **IG1:** 16 **IG2:** 18 **IG3:** 13 **CG:** 17	NR	NR	No numbers reported. Participants who were receiving 5‐ASA, corticosteroids, AZA or 6‐MP before the study continued to receive a stable dose during the trial period. The dose of corticosteroids could be tapered beginning 8 weeks after the initiation of the study. Treatment with these drugs or with MTX or ciclosporin could not be initiated during the trial.
Ye 2019	**IG:** CT‐P13 biosimilar 5 mg/kg **CG:** IFX 5 mg/kg at weeks 0, 2, 6, and then every 8 weeks up to week 54	None	**IG:** Steroids 7/111 (33%) AZA 84/111 (76%) **CG:** Steroids 33/109 (30%) AZA 80/109 (73%)	NR	NR	NR

5‐ASA: 5‐aminosalicylic acid; 6‐MP: 6‐mercaptopurine; AZA: azathioprine; cA2: early name for infliximab; CG: control group; CT‐P13: subcutaneous infliximab; IFX: infliximab; IG: intervention group; IV: intravenous; MTX: methotrexate; n: number; NR: not reported; PO: oral; qd: once daily; TNF: tumour necrosis factor.

**3:** Primary and secondary outcomes

**Study ID**	**Group interventions**	**Primary outcomes**			**Secondary outcomes**				
		**Clinical remission**	**Clinical response**	**Withdrawals due to adverse events**	**Endoscopic remission**	**Histological remission**	**Endoscopic response**	**Serious adverse events**	**Total adverse events**
Colombel 2010	**IG1:** IFX 5 mg/kg **IG2:** combination therapy **CG:** AZA 2.5 mg/kg	By week 26 (used in analysis) **IG1:** 81/169 **IG2:** 102/169 **CG:** 54/170 By week 50 **IG1:** 64/169 **IG2:** 80/169 **CG:** 41/170	CDAI 70 response (week 26) **IG1:** 95/169 **IG2:** 113/169 **CG:** 71/170 CDAI 70 response (week 50) **IG1:** 75/169 **IG2:** 88/169 **CG:** 56/170 CDAI 100 response (week 26) used in analysis **IG1:** 92/169 **IG2:** 105/169 **CG:** 64/170 CDAI 100 response (week 50) **IG1:** 70/169 **IG2:** 85/169 **CG:** 47/170	At week 54 **IG1:** 29/169 **IG2:** 37/169 **CG:** 42/170	Mucosal healing (week 26) **IG1:** 28/169 **IG2:** 47/169 **CG:** 18/170 *Randomised participants not assessed with endoscopy were counted as failures*	NR	NR	At week 54 **IG1:** 39/169 **IG2:** 27/169 **CG:** 43/170	Through week 30 **IG1:** 41/169 **IG2:** 39/169 **CG:** 63/170 Through week 50 **IG1:** 53/169 **IG2:** 53/169 **CG:** 69/170
D'Haens 1999	**IG1:** IFX 5 mg **IG2:** IFX 10 mg **IG3:** IFX 20 mg **CG:** placebo 0.1% human serum albumin	NR	NR as dichotomous CDAI mean at week 4 **IG1:** 122.8 (SEM 26.1) **IG2:** 220.5 (SEM 63.4) **IG3:** 161.9 (SEM 34.5) **CG:** 261.3 (SEM 33.3)	Unclear	NR	NR	NR as dichotomous CDEIS mean at week 4 **IG1:** 6.4 (SEM 5.1) **IG2:** 4.3 (SEM 5.4) **IG3:** 5.2 (SEM 2.8) **CG:** 7.5 (SEM 5.4)	NR (except for 1 infliximab‐treated participant with exclusive rectosigmoidal Crohn's disease developed a new rectal stricture at the site of earlier severe ulceration)	NR
D'Haens 2008	**IG:** IFX 5 mg/kg + AZA 2–2.5 mg/kg **CG:** corticosteroids	Week 14 **IG:** 43/67 **CG:** 21/66 (taken from graph) After this time, infliximab was given to the CG and therefore no further data reported	NR	**IG** 14/67 **CG** 14/66 at end of study	NR (only assessed post‐hoc)	NR	NR	**IG** 20/67 **CG** 19/66 *Not known at end of trial – we assumed that these occurred at 26 weeks*	Unclear – numbers not reported per participant
Duan 2013	**IG1:** IFX 5 mg/kg **IG2:** AZA 2.5 mg/kg+ IFX 5 mg/kg **CG:** AZA 2.5 mg/kg	Clinical remission at week 26 – decrease in CDAI < 70 points but a total CDAI score of < 150 **IG1:** 4/8 **IG2:** 5/8 **CG:** 3/8	Clinical efficacy at week 26 – decrease in CDAI ≥ 70 points or a decrease of ≥ 25% of the total CDAI score **CG:** 2/8 **IG1:** 2/8 **IG2:** 2/8	**IG1:** 1/8 **IG2:** 0/8 **CG:** 1/8	"Fully healed" **IG1:** 1/8 **IG2:** 4/8 **CG:** 3/8	NR	"Basically healing" **IG1:** 1/8 **IG2:** 1/8 **CG:** 1/8	NR	NR
Hanauer 2002	**IG1:** IFX infusion, regimen 1 **IG2:** IFX infusion, regimen 2 **CG:** placebo	NR	NR	NR	NR	NR	NR	NR	NR
Lemann 2006	**IG:** IFX 5 mg/kg **CG:** placebo	Week 12 **IG:** 43/57 **CG:** 22/58 Week 24 (primary) **IG:** 32/57 **CG:** 17/58 Week 52 **IG:** 23/57 **CG:** 13/58	NR	**IG:** 2/57 **CG:** 5/58	CDEIS Decrease from baseline (median): Week 24: **IG:** 6.9 (IQR 4.1 to 9.5) **CG:** 1.2 (IQR −1.5 to 4.4) Dichotomous outcome only reported for a subset	NR	NR	**IG:** 3/57 **CG:** 3/58 Table 2 described "Serious adverse event in 5 cases (infliximab, n = 3; placebo, n = 3)"	**IG:** 29/57 **CG:** 28/58
Present 1999	**IG1:** IFX 5 mg/kg **IG2:** IFX 10 mg/kg **CG:** placebo	Complete response (defined as the absence of any draining fistulas at 2 consecutive visit) **IG1:** 17/31 **IG2:** 12/32 **CG:** 4/31	A reduction of ≥ 50% from baseline in the number of draining fistulas observed at ≥ 2 consecutive study visits **IG1:** 21/31 **IG2:** 18/32 **CG:** 8/31	**IG1:** 1 **IG2:** 1 **CG:** 0	NR	NR	NR	5 in total **IG1:** 1/31 **IG2:** 4/32 **CG:** 0/31	**IG1:** 10/31 **IG2:** 26/32 **CG:** 12/31
Sands 2004b	**IG:** IFX 5 mg/kg **CG:** placebo	NR	**IG:** 9/43 **CG:** 7/44	NR	NR	NR	NR	NR	NR
Targan 1997	**IG1:** cA2 at 5 mg/kg **IG2:** cA2 at 10 mg/kg **IG3:** cA2 at 20 mg/kg **CG:** 0.1% human serum albumin	CDAI < 150 at week 4 (primary) **IG1:** 19/27 **IG2:** 11/28 **IG3:** 11/28 **CG:** 2/25 CDAI < 150 at week 2 **IG1:** 16/27 **IG2:** 10/28 **IG3:** 10/28 **CG:** 2/25	≥ 70‐point decrease clinical response at week 4 **IG1:** 22/27 **IG2:** 14/28 **IG3:** 18/28 **CG:** 4/25	Unclear	NR	NR	NR	Unclear	Unclear
Ye 2019	**IG:** CT‐P13 biosimilar 5 mg/kg **CG:** IFX 5 mg/kg	Clinical remission at weeks 6, 14, and 30 Week 6 (primary) **IG:** 47 **CG:** 49 Week 14 **IG:** 59 **CG:** 60 Week 30 **IG:** 61 **CG:** 62	Week 6 CDAI‐70 **IG:** 77 **CG:** 81 CDAI‐100 **IG:** 67 **CG:** 70 Week 14 CDAI‐70 **IG:** 96 **CG:** 96 CDAI‐100 **IG:** 78 **CG:** 83 Week 30 CDAI‐70 **IG:** 85 **CG:** 82 CDAI‐100 **IG:** 80 **CG:** 80	*Up to 30 weeks* **IG:** 17/111 **CG:** 21/109	NR	NR	NR	Up to 30 weeks **IG:** 6/111 **CG:** 9/109	Up to 30 weeks **IG:** 63/111 **CG:** 70/109

AZA: azathioprine; cA2: early name for infliximab; CDAI: Crohn's Disease Activity Index; CDEIS: Crohn's Disease Endoscopic Index of Severity; CG: control group; IFX: infliximab; IG: intervention group; IQR: interquartile range; NR: not reported; SEM: standard error of the mean.

##### Study design

Nine were large RCTs conducted across multicentre hospitals in Europe (Colombel 2010; D'Haens 1999; D'Haens 2008; Hanauer 2002; Lemann 2006; Present 1999; Sands 2004b; Targan 1997; Ye 2019). One was a single‐centre RCT conducted in China (Duan 2013). Ye 2019 was a cross‐over trial with cross‐over at week 30.

##### Interventions

- Colombel 2010: azathioprine 2.5 mg/kg compared to infliximab infusions of 5 mg/kg compared to combination oral azathioprine 2.5 mg/kg and infliximab infusions of 5 mg/kg (three arms)
- D'Haens 1999: infusions of placebo compared to infliximab infusions of 5 mg/kg compared to infliximab infusions of 10 mg/kg compared to infliximab infusions of 20 mg/kg (four arms)
- D'Haens 2008: corticosteroids compared to infusions of infliximab 5 mg/kg and azathioprine (two arms)
- Duan 2013: azathioprine compared to infusions of infliximab 5 mg/kg compared to azathioprine and infusions of infliximab 5 mg/kg (three arms)
- Hanauer 2002: placebo infusions compared to infusions of infliximab 5 mg/kg compared to infusions of infliximab 10 mg/kg (three arms). Only the non‐responders part of this trial was relevant for this review, as the responders were randomised separately for a maintenance trial.
- Lemann 2006: azathioprine compared to azathioprine and infusions of infliximab 5 mg/kg (two arms)
- Present 1999: placebo infusions compared to infliximab infusions of 5 mg/kg compared to infliximab infusions of 10 mg/kg (three arms)
- Sands 2004b: placebo compared to infliximab infusions of 5 mg/kg (two arms). Only the non‐responders part of this trial is relevant for this review, as the responders were randomised separately for a maintenance trial.
- Targan 1997: infusions of placebo compared to infliximab infusions of 5 mg/kg compared to infliximab infusions of 10 mg/kg compared to infliximab infusions of 20 mg/kg (four arms)
- Ye 2019: infliximab infusions of 5 mg/kg compared to CT‐P13 biosimilar infusions of 5 mg/kg (two pre‐cross‐over arms)

##### Concurrent therapies

Colombel 2010 allowed participants to use systemic steroids and D'Haens 2008 allowed participants the use of azathioprine and methotrexate. Present 1999 allowed participants to receive corticosteroids, purine analogues, aminosalicylates and antibiotics.

For Targan 1997, participants who were receiving mesalamine, corticosteroids, azathioprine or mercaptopurine before the study continued to receive a stable dose during the trial. Treatment with these drugs or with methotrexate or ciclosporin could not be initiated during the trial.

Hanauer 2002 allowed the continued use of 5‐aminosalicylates or antibiotics, corticosteroids, azathioprine and 6‐mercaptopurine, or methotrexate.

Sands 2004b permitted 5‐aminosalicylates, oral corticosteroids, azathioprine, mercaptopurine, mycophenolate mofetil, methotrexate and antibiotics.

D'Haens 1999, Lemann 2006, Ye 2019, and Duan 2013 did not mention the use of concurrent therapies in their studies.

##### Disease activity

Eight studies reported disease activity at the beginning of the study, which was a Crohn's Disease Activity Index (CDAI) score between 220 and 400 (Colombel 2010; D'Haens 1999; D'Haens 2008; Hanauer 2002; Lemann 2006; Present 1999; Targan 1997; Ye 2019).

In Sands 2004b, disease activity was at least 150 on the CDAI scale.

One study did not report disease activity at the beginning of the study (Duan 2013).

##### Disease duration

Colombel 2010 reported median disease duration for all included participants as 2.3 years.

D'Haens 2008 reported median disease duration for the participants in the combined immunosuppression arm was two years and 2.5 years for those in the conventional management arm.

In Hanauer 2002, median disease duration was 37 (interquartile range 30–46) years for all non‐responder participants.

Lemann 2006 reported a disease duration between five and seven years for the treatment‐failure cohort, and three to four years for the treatment‐naive cohort.

Present 1999 reported a disease duration for all participants of 11 to 13 years.

In Sands 2004b, disease duration ranged between 0.3 and 49.8 years for all non‐responder participants.

Four studies did not report disease duration (D'Haens 1999; Duan 2013; Targan 1997; Ye 2019).

##### Location of disease

Ninety‐one participants had ileal disease, 274 ileocolonic disease and 161 colonic disease (D'Haens 1999; D'Haens 2008; Lemann 2006; Present 1999; Targan 1997).

Lemann 2006 reported findings from 26 participants with active perianal disease.

Targan 1997 reported findings from 53 participants who had undergone previous segmental resection.

Present 1999 and Sands 2004b were performed with exclusively fistulating populations.

Targan 1997 and Duan 2013 did not report numbers of participants with fistulating disease.

Ye 2019 did not report disease location.

Colombel 2010 reported disease location; however, it is unclear what proportion of the population was in which category due to the nature of the reporting.

##### Age

All studies reported mean or median participant age, which was 26 to 65 years.

##### Conflicts of interest

All studies except Duan 2013 declared conflicts of interest and funding from pharmaceutical companies. It is unclear whether Duan 2013 had conflicts or not as there were no related statements. The full declarations can be found in the Characteristics of included studies tables.

#### Excluded studies

We excluded 47 studies (see Characteristics of excluded studies table).

- Twenty‐three for ineligible study design (Baima 2016; Bernstein 2002; Bernstein 2020; Billiet 2016; Blesl 2021; Bodini 2018; Bortlik 2013; Buhl 2020; Chaparro 2020; Colombel 2019; EUCTR2008‐006484‐36‐IT; EUCTR2011‐003038‐14‐NL; Fu 2011; Khanna 2015; Lichtenstein 2002; Narula 2021; NCT01442025; Sample 2002; Sanchez‐Hernandez 2020; Sands 2004c; Syversen 2020a; Syversen 2021; Yang 2015)
- Six for ineligible study intervention (Blumenstein 2006; EUCTR2021‐000469‐33‐NL; Faegan 2014; Mantzaris 2004; NCT04835506; Rutgeerts 1999)
- Eighteen for ineligible study population (Bossuyt 2019; Bossuyt 2021; D'Haens 2016; EUCTR2010‐018431‐18‐DE; Hao 2020; Jogensen 2019; Luna‐Chadid 2003; Mascheretti 2002; NCT00004941; NCT02883452; Ruemmele 2009; Rutgeerts 2005; Schroder 2006; Sorrentino 2012; Syversen 2020b; Szymanska 2016; Tajiri 2018; Yamamoto 2009)

#### Studies awaiting classification

No studies are awaiting classification.

#### Ongoing studies

One study is ongoing (KCT0007470; see Characteristics of ongoing studies table).

### Risk of bias in included studies

A summary of the risk of bias assessment is displayed in Figure 2.

**2:**
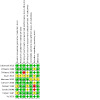
Risk of bias summary: review authors' judgements about each risk of bias item for each included study.

#### Allocation

Seven studies provided sufficient information about randomisation to judge them at low risk (Colombel 2010; D'Haens 1999; D'Haens 2008; Hanauer 2002; Lemann 2006; Sands 2004b; Ye 2019).

Three studies did not describe the randomisation method and so were judged at unclear risk (Duan 2013; Present 1999; Targan 1997).

Nine studies provided adequate description of allocation concealment and were judged at low risk (Colombel 2010; D'Haens 1999; D'Haens 2008; Hanauer 2002; Lemann 2006; Present 1999; Sands 2004b; Targan 1997; Ye 2019)

Duan 2013 was judged at unclear risk due to not providing sufficient information for judgement.

#### Blinding

Eight studies were blinded and judged at low risk of performance bias (Colombel 2010; D'Haens 1999; Hanauer 2002; Lemann 2006; Present 1999; Sands 2004b; Targan 1997; Ye 2019). D'Haens 2008 was at high risk as it was an open‐label study. Duan 2013 did not provide any information and was judged at unclear risk.

Six studies were blinded and judged at low risk for outcome assessment (Colombel 2010; D'Haens 1999; Hanauer 2002; Lemann 2006; Targan 1997; Ye 2019). Four studies were judged at unclear risk due to lack of information (D'Haens 2008; Duan 2013; Present 1999; Sands 2004b)

#### Incomplete outcome data

Nine studies were at low risk for attrition bias (Colombel 2010; D'Haens 1999; D'Haens 2008; Duan 2013; Lemann 2006; Present 1999; Sands 2004b; Targan 1997; Ye 2019).

Hanauer 2002 was at unclear risk.

#### Selective reporting

Three studies were at low risk for selective reporting bias (Colombel 2010; D'Haens 2008; Ye 2019), and another five at unclear risk (D'Haens 1999; Duan 2013; Hanauer 2002; Lemann 2006; Targan 1997).

Two studies were at high risk (Present 1999; Sands 2004b).

#### Other potential sources of bias

Nine studies were at low risk for other bias (Colombel 2010; D'Haens 1999; D'Haens 2008; Duan 2013; Lemann 2006; Present 1999; Sands 2004b; Targan 1997; Ye 2019).

Hanauer 2002 was at unclear risk.

### Effects of interventions

See: Table 1; Table 2; Table 3; Table 4; Table 5; Table 6; Table 7; Table 8; Table 9

#### Infliximab 5 mg/kg to 10 mg/kg versus placebo

Three studies compared infliximab to placebo on generalised CD populations (D'Haens 1999; Hanauer 2002; Targan 1997). Hanauer 2002 was on participants who did not respond to infliximab during the preliminary induction phase of RCTs assessing the effects of infliximab as maintenance therapy, and which randomised and followed up their non‐responder participants in parallel to the responders.

##### Primary outcome: achievement of clinical remission

Infliximab 5 mg/kg to 10 mg/kg may be more effective for clinical remission compared to placebo.

Targan 1997 reported 30/55 participants receiving combined infliximab 5 mg/kg and 10 mg/kg doses achieved clinical remission at week four compared to 3/25 participants in the placebo group (RR 4.55, 95% CI 1.53 to 13.50; NNTB 3, 95% 2 to 9; low‐certainty evidence; Analysis 1.1; Table 1).

We downgraded the certainty of the evidence one level due to serious concerns with imprecision and one level due to serious concerns risk of bias.

The other studies did not report this outcome (D'Haens 1999; Hanauer 2002).

##### Primary outcome: achievement of clinical response

Infliximab 5 mg/kg to 10 mg/kg may be more effective for clinical response compared to placebo.

Targan 1997 reported clinical response, defined as reduction of CDAI by 70 points or more at week four. This was achieved by 36/55 participants receiving 5 mg/kg and 10 mg/kg infliximab doses versus 4/24 participants in the placebo group (RR 4.09, 95% CI 1.63 to 10.25; NNTB 3, 95% CI 2 to 5; low‐certainty evidence; Analysis 1.2; Table 1).

D'Haens 1999 reported disease activity using the CDAI score at week four as a mean of 122.8 (SEM 26.1) for the 5 mg/kg group, 220.5 (SEM 63.4) for the 10 mg/kg group and 261.3 (SEM 33.3) for the placebo group.

We downgraded the certainty of the evidence one level due to serious concerns with imprecision and one level due to serious concerns with risk of bias.

Hanauer 2002 did not report any data for this outcome.

##### Primary outcome: withdrawal due to adverse events

No studies clearly reported withdrawal due to adverse events.

##### Secondary outcome: endoscopic remission

No studies clearly reported endoscopic remission.

##### Secondary outcome: histological remission

No studies reported histological remission.

##### Secondary outcome: endoscopic response

We could not draw any conclusions on the effects of infliximab compared to placebo for endoscopic remission.

D'Haens 1999 reported continuous endoscopic scores as a mean Crohn's Disease Endoscopic Index of Severity (CDEIS) scale score at week four. For the 5 mg/kg group this was 6.4 (SEM 5.1), for the 10 mg/kg group 4.3 (SEM 5.4), and for the placebo group 7.5 (SEM 5.4).

We downgraded the certainty of the evidence two levels due to very serious concerns with imprecision and one level due to serious concerns with risk of bias.

The remaining studies did not report this outcome.

##### Secondary outcome: serious adverse events

No studies sufficiently reported serious adverse events.

##### Secondary outcome: total adverse events

No studies sufficiently reported total adverse events.

#### Infliximab 5 mg/kg to 10 mg/kg versus placebo for exclusively fistulating population

Two studies compared infliximab to placebo for exclusively fistulating CD populations (Present 1999; Sands 2004b). Sands 2004b was on participants who did not respond to infliximab during the preliminary induction phase of RCTs assessing the effects of infliximab as maintenance therapy, and which randomised and followed up their non‐responder participants in parallel to the responders.

##### Primary outcome: achievement of clinical remission

We could not draw any conclusions on the effects of infliximab compared to placebo for clinical remission on exclusively fistulating participants.

Present 1999 reported 29/63 participants receiving infliximab 5 mg/kg and 10 mg/kg versus 4/31 participants receiving placebo achieved clinical remission defined as absence of any draining fistulas at consecutive visits (RR 3.57, 95% CI 1.38 to 9.25; NNTB 4, 95% CI 2 to 13; very low‐certainty evidence; Analysis 2.1; Table 2).

We downgraded the certainty of the evidence one level due to serious concerns with risk of bias and two levels due very serious concerns with imprecision.

Sands 2004b did not report this outcome.

##### Primary outcome: achievement of clinical response

We could not draw any conclusions on the effects of infliximab compared to placebo for clinical response on exclusively fistulating participants.

Both studies reported clinical response defined as reduction of 50% in the number of draining fistulas at two or more consecutive visits.

A total of 48/106 participants in the infliximab 5 mg/kg and 10 mg/kg groups versus 15/75 participants in the placebo group achieved clinical response (RR 1.94, 95% CI 1.10 to 3.41; I^2^ = 14%; NNTB 6, 95% CI 3 to 32; very low‐certainty evidence; Table 2).

We downgraded the certainty of the evidence one level due to serious concerns with risk of bias and two levels due to very serious concerns with imprecision.

##### Primary outcome: withdrawal due to adverse events

We could not draw any conclusions on the effects of infliximab compared to placebo on withdrawals due to adverse events on exclusively fistulating participants.

Present 1999 reported 2/63 participants withdrew due to adverse events in the infliximab 5 mg/kg and 10 mg/kg groups and 0/31 participants in the placebo group (RR 2.50, 95% CI 0.12 to 50.54; very low‐certainty evidence; Analysis 2.3; Table 2).

We downgraded the certainty of the evidence one level due to serious concerns with risk of bias and two levels due to very serious concerns with imprecision.

Sands 2004b did not report this outcome.

##### Secondary outcome: endoscopic remission

No studies reported endoscopic remission.

##### Secondary outcome: histological remission

No studies reported histological remission.

##### Secondary outcome: endoscopic response

No studies reported endoscopic response.

##### Secondary outcome: serious adverse events

We could not draw any conclusions on the effects of infliximab compared to placebo on serious adverse events on exclusively fistulating participants.

Present 1999 reported 5/63 serious adverse events in the infliximab 5 mg/kg and 10 mg/kg groups and 0/31 events in the placebo group (RR 5.50, 95% CI 0.31 to 96.40; very low‐certainty evidence; Analysis 2.4).

We downgraded the certainty of the evidence one level due to serious concerns with risk of bias, and two levels due to very serious concerns with imprecision.

Sands 2004b did not report this outcome.

##### Secondary outcome: total adverse events

We could not draw any conclusions on the effects of infliximab compared to placebo on total adverse events on exclusively fistulating participants.

Present 1999 reported a total of 36/63 participants with adverse events in the infliximab 5 mg/kg and 10 mg/kg groups and 12/31 participants in the placebo group (RR 1.48, 95% CI 0.90 to 2.41; very low‐certainty evidence; Analysis 2.5).

We downgraded the certainty of the evidence one level due to serious concerns with risk of bias and two levels due to very serious concerns with imprecision.

Sands 2004b did not report this outcome.

#### Infliximab 5 mg/kg combined with purine analogues versus purine analogues

Four studies compared infliximab combined with azathioprine or 6‐mercaptopurine (purine analogues) to purine analogues alone. Two studies compared a combination group (infliximab and purine analogues) to infliximab alone and to purine analogues alone (Colombel 2010; Duan 2013). One study compared infliximab plus purine analogues to placebo plus purine analogues (Lemann 2006). D'Haens 2008 also compared infliximab plus purine analogues to purine analogues alone. However, at week 14, they added infliximab to all participants in the purine analogues alone group (D'Haens 2008). Therefore, we used data prior to the addition of infliximab.

##### Primary outcome: achievement of clinical remission

All four studies were included in a meta‐analysis (Colombel 2010; D'Haens 2008; Duan 2013; Lemann 2006).

Infliximab used in combination with purine analogues is probably more effective at inducing remission in CD than purine analogues alone at 24 to 26 weeks (182/301 participants with infliximab plus purine analogues versus 95/302 participants with purine analogues alone; RR 1.92, 95% CI 1.59 to 2.32; I^2^ = 0%; NNTB 4, 95% 3 to 5; 4 studies; moderate‐certainty evidence; Analysis 3.1; Table 3). We downgraded the certainty of the evidence one level due to serious concerns with risk of bias.

A sensitivity analysis using the fixed‐effect model produced similar results (RR 1.93, 95% CI 1.58 to 2.35; Analysis 3.2).

A sensitivity analysis that considered only studies that included participants who were naive to biologicals produced similar results (RR 1.94, 95% CI 1.56 to 2.43; Analysis 3.3).

##### Primary outcome: achievement of clinical response

Two studies reported clinical response (Colombel 2010; Duan 2013).

Infliximab used in combination with purine analogues is probably more effective at inducing response in CD than purine analogues alone at week 26 (107/177 participants with infliximab plus purine analogues versus 66/178 participants with purine analogues alone; RR 1.64, 95% CI 1.31 to 2.05; I^2^ = 0%; NNTB 5, 95% CI 4 to 8; moderate‐certainty evidence; Analysis 3.4; Table 3).

We downgraded the certainty of the evidence one level due to serious concerns with risk of bias.

The other studies did not report this outcome clearly.

##### Primary outcome: withdrawals due to adverse events

Four studies contributed data for this outcome (Colombel 2010; D'Haens 2008; Duan 2013; Lemann 2006).

There may be little to no difference in the occurrence of withdrawals due to adverse events with infliximab and purine analogues compared to purine analogues alone (53/301 participants with infliximab plus purine analogues versus 62/302 participants with purine analogues alone; RR 0.87, 95% CI 0.63 to 1.21; low‐certainty evidence; Analysis 3.5; Table 3).

We downgraded the certainty of the evidence one level due to serious concerns with risk of bias and one level due to serious concerns with imprecision.

The other studies did not report this outcome.

##### Secondary outcome: endoscopic remission

Three studies reported endoscopic remission (Colombel 2010; D'Haens 2008; Lemann 2006).

Infliximab used in combination with purine analogues may be more effective at inducing endoscopic remission than when using purine analogues alone (51/177 participants with infliximab plus purine analogues versus 21/178 participants with purine analogues alone; RR 2.27, 95% CI 1.31 to 3.94; I^2^ = 15%; NNTB 6, 95% 4 to 15; low‐certainty evidence; Analysis 3.6).

We downgraded the certainty of the evidence one level due to serious concerns with risk of bias and one level due to serious concerns with imprecision.

Duan 2013 did not report this outcome.

##### Secondary outcome: histological remission

No studies reported histological remission.

##### Secondary outcome: endoscopic response

No studies reported endoscopic response.

##### Secondary outcome: serious adverse events

Three studies reported serious adverse events (Colombel 2010; D'Haens 2008; Lemann 2006).

Infliximab used in combination with purine analogues may be no different to purine analogues alone for serious adverse effects (50/293 participants with infliximab plus purine analogues versus 65/294 participants with purine analogues alone; RR 0.79, 95% CI 0.55 to 1.11; I^2^ = 7%; low‐certainty evidence; Analysis 3.7).

We downgraded the certainty of the evidence one level due to serious concerns with risk of bias and one level due to serious concerns with imprecision.

Duan 2013 did not report this outcome.

##### Secondary outcome: total adverse events

Two studies reported total adverse events (Colombel 2010; Lemann 2006).

Infliximab used in combination with purine analogues may be no different to purine analogues alone for total adverse effects (82/226 participants with infliximab plus purine analogues versus 97/228 participants with purine analogues alone; RR 0.88, 95% CI 0.65 to 1.20; I^2^ = 42%; low‐certainty evidence; Analysis 3.8).

We downgraded the certainty of the evidence one level due to serious concerns with risk of bias and one level due to serious concerns with imprecision.

The other studies did not report this outcome.

#### Infliximab 5 mg/kg versus purine analogues

Two studies compared infliximab 5 mg/kg versus purine analogues (Colombel 2010*;*Duan 2013).

##### Primary outcome: achievement of clinical remission

Both studies reported clinical remission.

Infliximab may be more effective at inducing remission than when using purine analogues (85/177 participants with infliximab versus 57/178 participants with purine analogues; RR 1.50, 95% CI 1.15 to 1.95; I^2^ = 0%; NNTB 7, 95% 4 to 19; low‐certainty evidence; Analysis 4.1; Table 4).

We downgraded the certainty of the evidence one level due to serious concerns with risk of bias and one level due to serious concerns with imprecision.

##### Primary outcome: achievement of clinical response

Data from both studies were included in a meta‐analysis.

Infliximab may be more effective at inducing clinical response than when using purine analogues at week 26 (94/177 participants with infliximab versus 66/178 participants with purine analogues; RR 1.44, 95% CI 1.13 to 1.82; I^2^ = 0%; NNTB 7, 95% 4 to 18; low‐certainty evidence; Analysis 4.2; Table 4).

We downgraded the certainty of the evidence one level due to serious concerns with risk of bias and one level due to serious concerns with imprecision.

##### Primary outcome: withdrawals due to adverse events

Data from both studies were included in a meta‐analysis.

Infliximab may be no different to purine analogues for withdrawals due to adverse events (30/177 participants with infliximab versus 43/178 participants with purine analogues; RR 0.70, 95% CI 0.46 to 1.06; I^2^ = 0%; low‐certainty evidence; Analysis 4.3; Table 4).

We downgraded the certainty of the evidence one level due to serious concerns with risk of bias and one level due to serious concerns with imprecision.

##### Secondary outcome: endoscopic remission

Data from both studies were included in a meta‐analysis.

Infliximab may be no different to purine analogues for achievement of endoscopic remission (29/177 participants with infliximab versus 21/178 participants with purine analogues; RR 1.00, 95% CI 0.25 to 3.96; I^2^ = 51%; low‐certainty evidence; Analysis 4.4).

We downgraded the certainty of the evidence one level due to serious concerns with risk of bias and one level due to serious concerns with imprecision.

##### Secondary outcome: histological remission

No studies reported histological remission.

##### Secondary outcome: endoscopic response

No studies reported endoscopic response.

##### Secondary outcome: serious adverse events

One study reported serious adverse events (Colombel 2010).

Infliximab may be no different to purine analogues for serious adverse events (39/169 participants with infliximab versus 43/170 participants with purine analogues; RR 0.91, 95% CI 0.63 to 1.33; low‐certainty evidence; Analysis 4.5).

We downgraded the certainty of the evidence two levels due to very serious concerns with imprecision.

Duan 2013 did not report this outcome.

##### Secondary outcome: total adverse events

One study reported total adverse events (Colombel 2010).

Infliximab may be no different to purine analogues for total adverse events (53/169 participants with infliximab versus 69/170 participants with purine analogues; RR 0.77, 95% CI 0.58 to 1.03; low‐certainty evidence; Analysis 4.6).

We downgraded the certainty of the evidence two levels due to very serious concerns with imprecision.

Duan 2013 did not report this outcome.

#### Infliximab 5 mg/kg versus infliximab 10 mg/kg

Three studies compared infliximab 5 mg/kg to infliximab 10 mg/kg in a generalised CD population (D'Haens 1999; Hanauer 2002; Targan 1997).

##### Primary outcome: achievement of clinical remission

We could not draw any conclusions on the effects of infliximab 5 mg/kg compared to infliximab 10 mg/kg for clinical remission.

Targan 1997 reported 19/27 participants receiving infliximab 5 mg/kg achieved clinical remission compared to 11/28 participants receiving infliximab 10 mg/kg (RR 1.79, 95% CI 1.06 to 3.02; NNTB 4, 95% CI 3 to 23; Analysis 5.1; Table 5).

D'Haens 1999 did not provide a dichotomous definition for remission. They reported a mean CDAI score of 261.3 (SEM 33.3) for the infliximab 5 mg/kg group (eight participants) and 122.8 (SEM 26.1) for the 10 mg/kg group (eight participants).

The evidence was very low certainty; we downgraded two levels due to very serious concerns with imprecision and one level due to serious concerns with risk of bias.

Hanauer 2002 did not report this outcome for their infliximab non‐responder participants.

##### Primary outcome: achievement of clinical response

We could not draw any conclusions on the effects of infliximab 5 mg/kg compared to 10 mg/kg for clinical response.

Targan 1997 reported 22/27 participants receiving infliximab 5 mg/kg achieved clinical response compared to 14/28 participants receiving infliximab 10 mg/kg (RR 1.63, 95% CI 1.08 to 2.46; NNTB 4, 95% CI 3 to 16; very low‐certainty evidence; Analysis 5.2; Table 5).

We downgraded the certainty of the evidence two levels due to very serious concerns with imprecision and one level due to serious concerns with risk of bias.

The other studies did not report this outcome.

##### Primary outcome: withdrawals due to adverse events

No studies clearly reported withdrawal due to adverse events.

##### Secondary outcome: endoscopic remission

We could not draw any conclusions on the effects of infliximab 5 mg/kg compared to 10 mg/kg for endoscopic remission.

D'Haens 1999 did not provide a dichotomous definition for endoscopic remission. They reported a mean CDEIS score of 6.4 (SEM 5.1) for the infliximab 5 mg/kg group (eight participants) and 4.3 (SEM 5.4) for the 10 mg/kg group (eight participants).

The evidence was very low certainty; we downgraded two levels due to very serious concerns with imprecision and one level due to serious concerns with risk of bias.

The other studies did not report this outcome.

##### Secondary outcome: histological remission

No studies reported histological remission.

##### Secondary outcome: endoscopic response

No studies reported endoscopic response.

##### Secondary outcome: serious adverse events

No studies clearly reported serious adverse events.

##### Secondary outcome: total adverse events

No studies clearly reported total adverse events.

#### Infliximab 5 mg/kg versus infliximab 10 mg/kg for exclusively fistulating population

One study compared infliximab 5 mg/kg to infliximab 10 mg/kg in an exclusively fistulating CD population (Present 1999).

##### Primary outcome: achievement of clinical remission

We could not draw any conclusions on the effects of infliximab 5 mg/kg compared to infliximab 10 mg/kg for remission in an exclusively fistulating population.

Present 1999 reported the remission rate was 17/31 in the infliximab 5 mg/kg group and 12/32 in the infliximab 10 mg/kg group (RR 1.46, 95% CI 0.84 to 2.53; very low‐certainty evidence; Analysis 6.1; Table 6).

We downgraded the certainty of the evidence two levels due to very serious concerns with imprecision and one level due to serious concerns with risk of bias.

##### Primary outcome: achievement of clinical response

We could not draw any conclusions on the effects of infliximab 5 mg/kg compared to infliximab 10 mg/kg for response in an exclusively fistulating population.

Present 1999 reported the response rate was 21/31 in the infliximab 5 mg/kg group and 18/32 in the infliximab 10 mg/kg group (RR 1.20, 95% CI 0.82 to 1.78; very low‐certainty evidence; Analysis 6.2; Table 6).

We downgraded the certainty of the evidence two levels due to very serious concerns with imprecision and one level due to serious concerns with risk of bias.

##### Primary outcome: withdrawals due to adverse events

We could not draw any conclusions on the effects of infliximab 5 mg/kg compared to infliximab 10 mg/kg on withdrawals due to adverse events in an exclusively fistulating population.

Present 1999 reported withdrawals due to adverse events as 1/31 in the 5 mg/kg group and 1/32 in the 10 mg/kg group (RR 1.03, 95% CI 0.07 to 15.79; very low‐certainty evidence; Analysis 6.3; Table 6).

We downgraded the certainty of the evidence two levels due to very serious concerns with imprecision and one level due to serious concerns with risk of bias.

##### Secondary outcome: endoscopic remission

No studies reported endoscopic remission.

##### Secondary outcome: histological remission

No studies reported histological remission.

##### Secondary outcome: endoscopic response

No studies reported endoscopic response.

##### Secondary outcome: serious adverse events

We could not draw any conclusions on the effects of infliximab 5 mg/kg compared to infliximab 10 mg/kg on serious adverse events in an exclusively fistulating population.

Present 1999 reported serious adverse events as 1/31 in the infliximab 5 mg/kg group and 4/32 in the infliximab 10 mg/kg group (RR 0.26, 95% CI 0.03 to 2.18; very low‐certainty evidence; Analysis 6.4; Table 6).

We downgraded the certainty of the evidence two levels due to very serious concerns with imprecision and one level due to serious concerns with risk of bias.

##### Secondary outcome: total adverse events

We could not draw any conclusions on the effects of infliximab 5 mg/kg compared to infliximab 10 mg/kg on total adverse events in an exclusively fistulating population.

Present 1999 reported total adverse events as 10/31 in the infliximab 5 mg/kg group and 26/32 in the infliximab 10 mg/kg group (RR 0.40, 95% CI 0.23 to 0.68; very low‐certainty evidence; Analysis 6.5; Table 6).

We downgraded the certainty of the evidence two levels due to very serious concerns with imprecision and one level due to serious concerns with risk of bias.

#### Infliximab 5 mg/kg versus infliximab 20 mg/kg

Two studies compared infliximab 5 mg/kg to infliximab 20 mg/kg (D'Haens 1999; Targan 1997)

##### Primary outcome: achievement of clinical remission

We could not draw any conclusions on the effects of infliximab 5 mg/kg compared to infliximab 20 mg/kg for clinical remission.

Targan 1997 reported 19/27 participants receiving infliximab 5 mg/kg achieved clinical remission compared to 11/28 participants receiving infliximab 20 mg/kg (RR 1.79, 95% CI 1.06 to 3.02; NNTB 4, 95% CI 3 to 23; Analysis 7.1; Table 7).

D'Haens 1999 did not provide a dichotomous definition for remission. They reported a mean CDAI score of 261.3 (SEM 33.3) for the infliximab 5 mg/kg group (eight participants) and 161.9 (SEM 34.5) for the 20 mg/kg group (eight participants).

The evidence was very low certainty; we downgraded two levels due to very serious concerns with imprecision and one level due to serious concerns with risk of bias.

##### Primary outcome: achievement of clinical response

We could not draw any conclusions on the effects of infliximab 5 mg/kg compared to infliximab 20 mg/kg for clinical response.

Targan 1997 reported 22/27 participants receiving infliximab 5 mg/kg achieved clinical response compared to 18/28 participants receiving infliximab 20 mg/kg (RR 1.27, 95% CI 0.91 to 1.76; very low‐certainty evidence; Analysis 7.2; Table 7).

We downgraded the certainty of the evidence two levels due to very serious concerns with imprecision and one level due to serious concerns with risk of bias.

The other studies did not report this outcome.

##### Primary outcome: withdrawals due to adverse events

No studies clearly reported withdrawals due to adverse events.

##### Secondary outcome: endoscopic remission

We could not draw any conclusions on the effects of infliximab 5 mg/kg compared to infliximab 20 mg/kg for endoscopic remission.

D'Haens 1999 did not provide a dichotomous definition for endoscopic remission. They reported a mean CDEIS score of 6.4 (SEM 5.1) for the infliximab 5 mg/kg group (eight participants) and 5.2 (SEM 2.8) for the infliximab 20 mg/kg group (eight participants).

The evidence was very low certainty; we downgraded two levels due to very serious concerns with imprecision and one level due to serious concerns with risk of bias.

The other studies did not report this outcome.

##### Secondary outcome: histological remission

No studies reported histological remission.

##### Secondary outcome: endoscopic response

No studies reported endoscopic response.

##### Secondary outcome: serious adverse events

No studies clearly reported serious adverse events.

##### Secondary outcome: total adverse events

No studies clearly reported total adverse events.

#### Infliximab 10 mg/kg versus infliximab 20 mg/kg

Two studies compared infliximab 10 mg/kg to infliximab 20 mg/kg (D'Haens 1999; Targan 1997).

##### Primary outcome: achievement of clinical remission

We could not draw any conclusions on the effects of infliximab 10 mg/kg compared to infliximab 20 mg/kg for clinical remission.

Targan 1997 reported 11/28 participants receiving infliximab 10 mg/kg achieved clinical remission compared to 11/28 participants receiving infliximab 20 mg/kg (RR 1.00, 95% CI 0.52 to 1.92; Analysis 8.1; Table 8).

D'Haens 1999 did not provide a dichotomous definition for remission. They reported a mean CDAI score of 220.5 (SEM 63.4) for the infliximab 10 mg/kg group (eight participants) and 161.9 (SEM 34.5) for the infliximab 20 mg/kg group (eight participants).

The evidence was very low certainty; we downgraded two levels due to very serious concerns with imprecision and one level due to serious concerns with risk of bias.

##### Primary outcome: achievement of clinical response

We could not draw any conclusions on the effects of infliximab 10 mg/kg compared to infliximab 20 mg/kg for clinical response.

Targan 1997 reported 14/28 participants receiving infliximab 5 mg/kg achieved clinical response compared to 18/28 receiving infliximab 20 mg/kg (RR 0.78, 95% CI 0.49 to 1.23; very low‐certainty evidence; Analysis 8.2; Table 8).

We downgraded the certainty of the evidence two levels due to very serious concerns with imprecision and one level due to serious concerns with risk of bias.

The other studies did not report this outcome.

##### Primary outcome: withdrawals due to adverse events

No studies clearly reported withdrawals due to adverse events.

##### Secondary outcome: endoscopic remission

We could not draw any conclusions on the effects of infliximab 10 mg/kg compared to infliximab 20 mg/kg for endoscopic remission.

D'Haens 1999 did not provide a dichotomous definition for endoscopic remission. They reported a mean CDEIS score of 4.3 (SEM 5.4) for the infliximab 10 mg/kg group (eight participants) and 5.2 (SEM 2.8) for the infliximab 20 mg/kg group (eight participants).

The evidence was of very low certainty; we downgraded two levels due to very serious concerns with imprecision and one level due to serious concerns with risk of bias.

The other studies did not report this outcome.

##### Secondary outcome: histological remission

No studies reported histological remission.

##### Secondary outcome: endoscopic response

No studies reported endoscopic response.

##### Secondary outcome: serious adverse events

No studies clearly reported serious adverse events.

##### Secondary outcome: total adverse events

No studies clearly reported total adverse events.

#### Infliximab 5 mg/kg versus CT‐P13 biosimilar at 5 mg/kg

Only one study compared infliximab to a biosimilar (Ye 2019).

##### Primary outcome: achievement of clinical remission

Infliximab may be equal to the biosimilar for the achievement of clinical remission (47/109 participants with infliximab 5 mg/kg versus 49/111 participants with biosimilar; RR 0.98, 95% CI 0.72 to 1.32; low‐certainty evidence; Analysis 9.1; Table 9).

We downgraded the certainty of the evidence two levels due to very serious imprecision.

##### Primary outcome: achievement of clinical response

Infliximab may be equal to the biosimilar for the achievement of clinical response (67/109 participants with infliximab 5 mg/kg versus 70/111 participants with biosimilar; RR 0.97, 95% CI 0.79 to 1.20; low‐certainty evidence; Analysis 9.2; Table 9).

We downgraded the certainty of the evidence two levels due to very serious imprecision.

##### Primary outcome: withdrawals due to adverse events

Infliximab may be similar to the biosimilar for withdrawals due to adverse events (21/109 participants with infliximab 5 mg/kg versus 17/111 participants with biosimilar; RR 1.26, 95% CI 0.70 to 2.25; low‐certainty evidence; Analysis 9.3; Table 9).

We downgraded the certainty of the evidence two levels due to very serious imprecision.

##### Secondary outcome: endoscopic remission

No studies reported endoscopic remission.

##### Secondary outcome: histological remission

No studies reported histological remission.

##### Secondary outcome: endoscopic response

No studies reported endoscopic response.

##### Secondary outcome: serious adverse events

Infliximab may be similar to the biosimilar for serious adverse events (9/109 participants with infliximab 5 mg/kg versus 6/111 participants with biosimilar; RR 1.53, 95% CI 0.56 to 4.15; low‐certainty evidence; Analysis 9.4).

We downgraded the certainty of the evidence two levels due to very serious imprecision.

##### Secondary outcome: total adverse events

Infliximab may be similar to the biosimilar for total adverse events (70/109 participants with infliximab 5 mg/kg versus 93/111 participants with biosimilar; RR 1.13, 95% CI 0.91 to 1.40; low‐certainty evidence; Analysis 9.5).

We downgraded the certainty of the evidence two levels due to very serious imprecision.

## Discussion

### Summary of main results

This review included 10 RCTs with 1101 randomised participants.

Infliximab may be more effective for inducing clinical remission and response than placebo.

Infliximab used in combination with purine analogues is probably more effective for inducing clinical remission than purine analogues alone (NNTB 4) and for inducing clinical response (NNTB 5). There may be little or no difference in the occurrence of withdrawals due to adverse events, the number of serious adverse events and the total number of adverse events. Infliximab and purine analogues combined may be more effective for endoscopic remission than purine analogues alone (NNTB 6).

Infliximab alone may be more effective for inducing clinical remission (NNTB 7) and response (NNTB 7) than purine analogues alone. There may be little or no difference in withdrawals due to adverse events, the number of serious adverse events and the total number of adverse events. There may be little or no difference between infliximab alone and purine analogues alone for endoscopic remission.

There may be little or no difference between infliximab and a CT‐P13 biosimilar for inducing clinical remission, clinical response and withdrawals due to adverse events.

We could not draw any conclusions when comparing infliximab 5 mg/kg to 10 mg/kg, 5 mg/kg to 20 mg/kg and 10 mg/kg to 20 mg/kg, for clinical remission or response, in a general CD population, because the certainty of the evidence was very low.

We could not draw any conclusions for all outcomes in the exclusively fistulating studies, including for the comparison of infliximab 5 mg/kg to 10 mg/kg compared to placebo.

We could not draw any conclusions for other outcomes because the available evidence was of very low certainty, or had not been reported.

### Overall completeness and applicability of evidence

The evidence is incomplete for several reasons. Given that the studies spanned 23 years between the first included studies (D'Haens 1999; Present 1999) and the latest study (Ye 2019), there have been changes in practice. These are associated with changes in definition of the disease and response that have contributed to heterogeneity of the patient populations and outcome measures employed that all limit the applicability of the evidence synthesised.

Most studies included in this review used the CDAI to recruit participants to studies and assessed clinical remission and response at different endpoints, which is considered the standard tool in CD research. However, other measures of disease state and in turn endpoints for studies, such as endoscopic and histological measures, were limited in reporting.

Moreover, in some contexts within CD, homogeneity of participants at entry is perhaps implicit, such as after surgery (Gjuladin‐Hellon 2019a; Gjuladin‐Hellon 2019b; Iheozor‐Ejiofor 2019). The studies included in this review involved people with different pre‐recruitment experience of therapy, as well as length and severity of disease. It has been demonstrated that a top‐down approach involving early biological and immunosuppressant use can lead to better results than using these in non‐responders (Baert 2010; D'Haens 2008). The disease state of participants and prior experience are key clinical factors that cannot be commented on due to the heterogeneity of the populations included in the primary studies.

This review found that trials mostly compared infliximab to a placebo or active comparator. However, with the increasing use of other biological drugs, the evidence in this area of practice is lacking.

The reporting of adverse events is another area of concern. It is common to experience difficulties in reporting related to heterogeneity of thresholds in defining serious or severe events, and as such, withdrawals are often the most available measure. For common effects, RCT data can be sufficient to consider safety (Gordon 2021a), but this is not the case for rarer and possibly more devastating outcomes where long‐term safety data are not addressed by this synthesis.

Finally, sample size of trials has resulted in issues with precision in most GRADE analyses. This is a pervasive issue within the field (Iheozor‐Ejiofor 2021).

This review did not include comparisons of routes of administration for infliximab, such as intravenous compared to subcutaneous. Future updates of this review will consider investigating this.

### Quality of the evidence

There were issues with unclear bias due to selective reporting. The older studies rarely reported protocols or registered trials and impacted the certainty of most GRADE judgements.

The certainty of outcomes on GRADE analysis range from moderate to very low, with the impact of both risk of bias and imprecision as key factors impacting the certainty of the evidence. Reporting of adverse events was also very sparse and so this was reflected in the GRADE analysis.

### Potential biases in the review process

Gaps in information to judge risk of bias was pervasive. We chose to contact the study authors for clarification or additional information; however, not all authors responded. We aim to include the data that may become available in future updates, but this could represent a source of bias in the review.

One study published the abstract in English while the full‐text article was in Chinese. We translated the study electronically as we were unable to find any contact information for the author group, so this may lead to a reporting bias (Duan 2013).

We are aware of the possibility of industry funding impacting the validity of the results. Funding from manufacturing companies or any conflicts of interests from both primary studies and the review team have been reported.

To our knowledge, this is the first Cochrane Review to specifically study the induction phase of infliximab and explicitly consider concurrent therapy with purine analogues. In some studies, this was the explicit goal of the study, but in others, the presence of such therapy (or indeed its absence) was only mentioned in a cursory manner. We believe this reflects a highly important clinical factor and source of heterogeneity and have, therefore, categorised studies as accurately as possible. However, this categorisation could be considered a source of bias.

### Agreements and disagreements with other studies or reviews

The previous Cochrane Review that assessed the overall efficacy and safety of TNF‐α antagonists had similar results showing the effectiveness of anti‐TNF agents in inducing remission in CD (Akobeng 2003).

The results broadly support current international guidelines in the UK (Lamb 2019), Europe (Torres 2020), and North America (Feuerstein 2021). However, the GRADE certainty ratings in these guidelines are higher than the judgements in this review.

## Authors' conclusions

**Implications for practice**

Infliximab alone may be more effective in inducing clinical remission and response than placebo (low‐certainty evidence).

Infliximab in combination with purine analogues is probably more effective than purine analogues alone in inducing clinical remission (moderate‐certainty evidence) and clinical response (moderate‐certainty evidence).

Infliximab alone may be more effective in inducing clinical remission and response than purine analogues alone (low‐certainty evidence).

Infliximab may be similar in efficacy to the CT‐P13 biosimilar and there may be little or no difference in withdrawals due to adverse events.

We were unable to draw meaningful conclusions whether infliximab alone is effective when used for exclusively fistulating populations.

There was evidence of little or no difference in withdrawal due to adverse events between infliximab and purines compared with purines alone, as well as infliximab alone compared with purines alone. Meaningful conclusions cannot be drawn on all other outcomes related to adverse events due to very low‐certainty evidence.

**Implications for research**

There does not appear to be a role for further studies comparing infliximab with placebo. Whilst the certainty of such outcomes is low, this is not a clinically meaningful comparison in research or practice. Rather, further targeted and appropriately designed randomised controlled trials may be needed to address the gaps in the evidence base in relation to active comparators. It is key that concurrent therapies and prior exposure to biological therapies are considered in the recruitment and design of studies and that these are clearly reported.

Other key future research would be comprehensive reporting on the effects of infliximab on endoscopic and histological remission, as these outcomes are rarely reported.

Appropriate powering and design of these studies based on appropriate minimum clinical difference data is needed to solve the issue with imprecision in outcomes and add more certainty to the evolving evidence base (Gordon 2021b).

Safety will always be a real priority but may need other design types and, in turn, other designs of synthesis, such as those using large cohort observational studies.

## What's new

**Table:** 

**Date**	**Event**	**Description**
4 December 2023	Amended	Author byline corrected.

## History

Protocol first published: Issue 4, 2017 Review first published: Issue 11, 2023

## Appendices

### Appendix 1. Search strategies

#### CENTRAL

Date run: 4 March 2023

#1 ([mh "Crohn Disease"] OR [mh ^"Inflammatory Bowel Diseases"] OR Crohn* OR Inflammatory Bowel Disease* OR IBD) AND ([mh Infliximab] OR Infliximab OR "ABP 710" OR ABP710 OR Avakine OR Avsola OR cA2 OR Flixabi OR "GP 1111" OR GP1111 OR IFX OR Inflectra OR Ixifi OR "PF 06438179" OR "PF 6438179" OR PF06438179 OR PF6438179 OR Remicade OR Remsima OR Renflexis OR Revellex OR "TA 650" OR TA650 OR Zessly) with Cochrane Library publication date Between Aug 2021 and Mar 2023, in Trials 83

#### Embase via OvidSP

Database: Embase <1974 to 2023 week 08>

1 exp Crohn Disease/ or Inflammatory Bowel Disease/ or (Crohn* or Inflammatory Bowel Disease* or IBD).mp. (199978)

2 Infliximab/ or (Infliximab or "ABP 710" or ABP710 or Avakine or Avsola or cA2 or Flixabi or "GP 1111" or GP1111 or IFX or Inflectra or Ixifi or "PF 06438179" or "PF 6438179" or PF06438179 or PF6438179 or Remicade or Remsima or Renflexis or Revellex or "TA 650" or TA650 or Zessly).mp. (167019)

3 Randomized controlled trial/ or Controlled clinical study/ or randomization/ or intermethod comparison/ or double blind procedure/ or human experiment/ or (random$ or placebo or (open adj label) or ((double or single or doubly or singly) adj (blind or blinded or blindly)) or parallel group$1 or crossover or cross over or ((assign$ or match or matched or allocation) adj5 (alternate or group$1 or intervention$1 or patient$1 or subject$1 or participant$1)) or assigned or allocated or (controlled adj7 (study or design or trial)) or volunteer or volunteers).ti,ab. or (compare or compared or comparison or trial).ti. or ((evaluated or evaluate or evaluating or assessed or assess) and (compare or compared or comparing or comparison)).ab. (6208790)

4 (random$ adj sampl$ adj7 ("cross section$" or questionnaire$1 or survey$ or database$1)).ti,ab. not (comparative study/ or controlled study/ or randomi?ed controlled.ti,ab. or randomly assigned.ti,ab.) (9365)

5 Cross‐sectional study/ not (randomized controlled trial/ or controlled clinical study/ or controlled study/ or (randomi?ed controlled or control group$1).ti,ab.) (338867)

6 (((case adj control$) and random$) not randomi?ed controlled).ti,ab. (21248)

7 (Systematic review not (trial or study)).ti. (251119)

8 (nonrandom$ not random$).ti,ab. (18726)

9 ("Random field$" or (random cluster adj3 sampl$)).ti,ab. (4426)

10 (review.ab. and review.pt.) not trial.ti. (1090581)

11 "we searched".ab. and (review.ti. or review.pt.) (48360)

12 ("update review" or (databases adj4 searched)).ab. (60294)

13 (rat or rats or mouse or mice or swine or porcine or murine or sheep or lambs or pigs or piglets or rabbit or rabbits or cat or cats or dog or dogs or cattle or bovine or monkey or monkeys or trout or marmoset$1).ti. and animal experiment/ (1214071)

14 Animal experiment/ not (human experiment/ or human/) (2549714)

15 or/4‐14 (4261110)

16 3 not 15 (5483547)

17 1 and 2 and 16 (5161)

18 limit 17 to embase (2352)

19 limit 18 to em=202134‐202308 (277)

#### MEDLINE via OvidSP

Database: Ovid MEDLINE(R) ALL <1946 to 2 March 2023>

1 Crohn Disease/ or Inflammatory Bowel Diseases/ or (Crohn* or Inflammatory Bowel Disease* or IBD).mp. (109450)

2 Infliximab/ or (Infliximab or "ABP 710" or ABP710 or Avakine or Avsola or cA2 or Flixabi or "GP 1111" or GP1111 or IFX or Inflectra or Ixifi or "PF 06438179" or "PF 6438179" or PF06438179 or PF6438179 or Remicade or Remsima or Renflexis or Revellex or "TA 650" or TA650 or Zessly).mp. (179114)

3 ((Randomized Controlled Trial or Controlled Clinical Trial).pt. or (Randomi?ed or Placebo or Randomly or Trial or Groups).ab. or Drug Therapy.fs.) not (exp Animals/ not Humans.sh.) (4899157)

4 1 and 2 and 3 (5100)

5 limit 4 to ed=20210831‐20230302 (501)

6 limit 4 to dt=20210831‐20230302 (321)

7 5 or 6 (543)

#### ClinicalTrials.gov

**Advanced search**

*Condition or disease:* Crohn Disease OR Inflammatory Bowel Diseases

*Study type:* Interventional Studies (Clinical Trials)

*Intervention/treatment:* Infliximab

*First posted from* 08/31/2021 *to* 03/04/2023

13 Studies found

#### WHO ICTRP

**Advanced search**

Crohn Disease OR Inflammatory Bowel Diseases *in the Condition*

Infliximab *in the Intervention*

*Recruitment Status is* ALL

*Date of registration is between* 01/01/2021 *and* 04/03/2023

14 records for 14 trials found

## Data and analyses

**Comparison 1:** Infliximab 5–10 mg/kg versus placebo

Outcome or subgroup title	No. of studies	No. of participants	Statistical method	Effect size
1.1 Clinical remission defined as CDAI < 150 by week 4	1	80	Risk Ratio (M‐H, Random, 95% CI)	4.55 [1.53, 13.50]
1.2 Clinical response defined as reduction of CDAI score by ≥ 70 at week 4	1	80	Risk Ratio (M‐H, Random, 95% CI)	4.09 [1.63, 10.25]

**Comparison 2:** Infliximab 5–10 mg/kg versus placebo for exclusively fistulating population

Outcome or subgroup title	No. of studies	No. of participants	Statistical method	Effect size
2.1 Clinical remission defined as absence of any draining fistulas at consecutive visits	1	94	Risk Ratio (M‐H, Random, 95% CI)	3.57 [1.38, 9.25]
2.2 Clinical response defined as reduction of 50% in the number of draining fistulas at ≥ 2 consecutive visits	2	181	Risk Ratio (M‐H, Random, 95% CI)	1.94 [1.10, 3.41]
2.3 Withdrawals due to adverse events	1	94	Risk Ratio (M‐H, Random, 95% CI)	2.50 [0.12, 50.54]
2.4 Serious adverse events	1	94	Risk Ratio (M‐H, Random, 95% CI)	5.50 [0.31, 96.40]
2.5 Total adverse events	1	94	Risk Ratio (M‐H, Random, 95% CI)	1.48 [0.90, 2.41]

**Comparison 3:** Infliximab 5 mg/kg and purine analogues versus purine analogues

Outcome or subgroup title	No. of studies	No. of participants	Statistical method	Effect size
3.1 Clinical remission as defined by the studies at weeks 24–26	4	603	Risk Ratio (M‐H, Random, 95% CI)	1.92 [1.59, 2.32]
3.2 Clinical remission as defined by the studies (sensitivity analysis for fixed effects)	4	603	Risk Ratio (M‐H, Fixed, 95% CI)	1.92 [1.59, 2.32]
3.3 Clinical remission as defined by the studies (sensitivity analysis for biologically naive patients)	2	472	Risk Ratio (M‐H, Random, 95% CI)	1.93 [1.56, 2.39]
3.4 Clinical response as defined by the studies at week 26	2	355	Risk Ratio (M‐H, Random, 95% CI)	1.64 [1.31, 2.05]
3.5 Withdrawals due to adverse events	4	603	Risk Ratio (M‐H, Random, 95% CI)	0.87 [0.63, 1.21]
3.6 Endoscopic remission as defined by the studies	2	355	Risk Ratio (M‐H, Random, 95% CI)	2.27 [1.31, 3.94]
3.7 Serious adverse events	3	587	Risk Ratio (M‐H, Random, 95% CI)	0.79 [0.55, 1.11]
3.8 Total adverse events	2	454	Risk Ratio (M‐H, Random, 95% CI)	0.88 [0.65, 1.20]

**Comparison 4:** Infliximab 5 mg/kg versus purine analogues

Outcome or subgroup title	No. of studies	No. of participants	Statistical method	Effect size
4.1 Clinical remission at week 26	2	355	Risk Ratio (M‐H, Random, 95% CI)	1.50 [1.15, 1.95]
4.2 Clinical response at week 26	2	355	Risk Ratio (M‐H, Random, 95% CI)	1.44 [1.13, 1.82]
4.3 Withdrawals due to adverse events	2	355	Risk Ratio (M‐H, Random, 95% CI)	0.70 [0.46, 1.06]
4.4 Endoscopic remission	2	355	Risk Ratio (M‐H, Random, 95% CI)	1.00 [0.25, 3.96]
4.5 Serious adverse events	1	339	Risk Ratio (M‐H, Random, 95% CI)	0.91 [0.63, 1.33]
4.6 Total adverse events	1	339	Risk Ratio (M‐H, Random, 95% CI)	0.77 [0.58, 1.03]

**Comparison 5:** Infliximab 5 mg/kg versus infliximab 10 mg/kg

Outcome or subgroup title	No. of studies	No. of participants	Statistical method	Effect size
5.1 Clinical remission defined as CDAI < 150 at week 4	1	55	Risk Ratio (M‐H, Random, 95% CI)	1.79 [1.06, 3.02]
5.2 Clinical response defined as reduction in CDAI by 70 points at week 4	1	55	Risk Ratio (M‐H, Random, 95% CI)	1.63 [1.08, 2.46]

**Comparison 6:** Infliximab 5 mg/kg versus infliximab 10 mg/kg for exclusively fistulating population

Outcome or subgroup title	No. of studies	No. of participants	Statistical method	Effect size
6.1 Clinical remission defined by closure of all fistulae	1	63	Risk Ratio (M‐H, Random, 95% CI)	1.46 [0.84, 2.53]
6.2 Clinical response defined as reduction of 50% in the number of draining fistulas at ≥ 2 consecutive visits	1	63	Risk Ratio (M‐H, Random, 95% CI)	1.20 [0.82, 1.78]
6.3 Withdrawals due to adverse events	1	63	Risk Ratio (M‐H, Random, 95% CI)	1.03 [0.07, 15.79]
6.4 Serious adverse events	1	63	Risk Ratio (M‐H, Random, 95% CI)	0.26 [0.03, 2.18]
6.5 Total adverse events	1	63	Risk Ratio (M‐H, Random, 95% CI)	0.40 [0.23, 0.68]

**Comparison 7:** Infliximab 5 mg/kg versus infliximab 20 mg/kg

Outcome or subgroup title	No. of studies	No. of participants	Statistical method	Effect size
7.1 Clinical remission defined as CDAI < 150 at week 4	1	55	Risk Ratio (M‐H, Random, 95% CI)	1.79 [1.06, 3.02]
7.2 Clinical response defined as reduction of CDAI score by ≥ 70 at week 4	1	55	Risk Ratio (M‐H, Random, 95% CI)	1.27 [0.91, 1.76]

**Comparison 8:** Infliximab 10 mg/kg versus infliximab 20 mg/kg

Outcome or subgroup title	No. of studies	No. of participants	Statistical method	Effect size
8.1 Clinical remission defined as CDAI < 150 at week 4	1	56	Risk Ratio (M‐H, Random, 95% CI)	1.00 [0.52, 1.92]
8.2 Clinical response defined as reduction of CDAI score by ≥ 70 at week 4	1	56	Risk Ratio (M‐H, Random, 95% CI)	0.78 [0.49, 1.23]

**Comparison 9:** Infliximab 5 mg/kg versus biosimilar 5 mg/kg

Outcome or subgroup title	No. of studies	No. of participants	Statistical method	Effect size
9.1 Clinical remission defined as CDAI < 150 at week 6	1	220	Risk Ratio (M‐H, Random, 95% CI)	0.98 [0.72, 1.32]
9.2 Clinical response defined as reduction of CDAI score by ≥ 100 at week 6	1	220	Risk Ratio (M‐H, Random, 95% CI)	0.97 [0.79, 1.20]
9.3 Withdrawals due to adverse events	1	220	Risk Ratio (M‐H, Random, 95% CI)	1.26 [0.70, 2.25]
9.4 Serious adverse events	1	220	Risk Ratio (M‐H, Random, 95% CI)	1.53 [0.56, 4.15]
9.5 Total adverse events	1	220	Risk Ratio (M‐H, Random, 95% CI)	1.13 [0.91, 1.40]

## Characteristics of studies

### Characteristics of included studies [ordered by study ID]

**Table:** Colombel 2010

***Study characteristics***		
Methods	**Study design:** 3‐arm parallel RCT **Number of centres:** 92 centres **Countries:** multiple **Study chronology:** March 2005 to November 2008 **Setting:** NR	
Participants	**Inclusion criteria** Diagnosis of CD for ≥ 6 weeks Moderate‐to‐severe disease activity (CDAI ≥ 220 and ≤ 450) No history of azathioprine, 6‐MP or biological treatments Are either: corticosteroid‐dependent, OR considered for a 2nd (or greater) course of corticosteroid, OR 5‐ASA failures, OR budesonide failures **Exclusion criteria** History of abdominal surgery within last 6 months Have an ostomy or stoma (an operation to create an opening from an area inside the body to the outside) Pregnant, breastfeeding or planning pregnancy (both men and women) Serious simultaneous illness that could interfere with study participation Use of any investigational drug within 30 days Have a concomitant diagnosis or any history of congestive heart failure Weigh > 140 kg (or 310 pounds) **Baseline disease characteristics** Fistulating disease: NR Location of disease (ileal, colonic, etc.): AZA group (n = 170): ileal or colon: 170/170 (100%), ileum only: 68/170 (40.0%), colon only: 33/170 (19.4%), ileum and colon: 69/170 (40.6%), proximal gastrointestinal tract: 7/170 (4.1%) IFX group (n = 169): ileal or colon: 163/169 (96.4%), ileum only: 54/163 (33.1%), colon only: 45/163 (27.6%), ileum and colon: 64/163 (39.3%), proximal gastrointestinal tract: 12/169 (7.1%) Combination (AZA + IFX) therapy group (n = 169): ileal or colon: 167/169 (98.8%), ileum only: 54/167 (32.2%), colon only: 40/167 (24.0%), ileum and colon: 73/167 (43.7%), proximal gastrointestinal tract: 16/169 (9.5%) All participants (n = 508): ileum or colon: 500/508 (98.4%), ileum only: 176/500 (35.2%), colon only: 118/500 (23.6%), ileum and colon: 206/500 (41.2%), proximal gastrointestinal tract: 35/508 (6.9%) Duration or length of disease since diagnosis: all participants included had to have ≥ 6 weeks of CD to be eligible **Active disease characteristics** Disease activity score: CDAI: AZA group (n = 170): 287.2 (SD 52.9), IFX group (n = 169): 284.8 (SD 62.1), combination therapy group (n = 169): 289.9 (SD 55.0), all participants (n = 508): 287.3 (SD 56.7) Length of active disease: median disease duration (years): AZA group: 2.4, IFX group: 2.2, combination therapy group: 2.2, all participants: 2.3 Endoscopic disease activity scoring: NR **Age at beginning of study** Median age (years): AZA group: 35.0 (range 18–79), IFX group: 35.0 (range 18–80), combination therapy group: 34.0 (range 19–68), all participants: 34.0 (range 18–80) **On 27 March 2007, after hepatosplenic T‐cell lymphoma had been reported in adolescents and very young adults receiving combination therapy with AZA and IFX, the protocol was amended to increase the minimum eligible age from 18 to 21 years. **Sex (male/female)** Male: AZA group (n = 170): 90 (52.9%), IFX group (n = 169): 84 (49.7%), combination therapy group (n = 169): 88 (52.7%), all participants (n = 508): 262 (51.6%) Female: AZA group (n = 170): 80 (47.1%), IFX group (n = 169): 85 (50.3%), combination therapy group (n = 169): 81 (47.9%), all participants (n = 508): 246 (48.4%) *(calculated from male data)* **Smoking:** NR	
Interventions	**IG1:** IFX only; IV infusion of IFX 5 mg/kg bodyweight at weeks 0, 2, 6, 14 and 22 plus daily oral placebo capsules to 30 weeks **IG2:** combination therapy; IV infusion IFX 5 mg/kg at weeks 0, 2, 6, 14 and 22 plus daily oral capsules 2.5 mg/kg of bodyweight to 30 weeks **CG1:** AZA only group; daily oral capsules 2.5 mg/kg bodyweight to 30 weeks plus placebo infusion at weeks 0, 2, 6, 14 and 22 **Duration of study:** 30 weeks **Measurement time points during study:** participants with corticosteroid‐free clinical remission at weeks 6, 10, 18 and 26 **Follow‐up measurements after study end:** measuring participants with week 26 status – weeks 34, 42 and 50	
Outcomes	**Primary outcomes as defined by study authors** Rate of corticosteroid‐free clinical remission at week 26 **Secondary outcomes as defined by study authors** Rates of corticosteroid‐free clinical remission at other time points Mucosal healing at week 26 amongst those who had ulcerations at baseline Rate of any remission Response‐70 Response‐100 IBDQ score Corticosteroid dose at each data‐collection time point (weeks 0, 2, 6, 10, 18, 26, 34, 42 and 50) Change in the CRP level from baseline to week 26	
Notes	**Funding source:** research grants from Centocor Ortho Biotech and Schering‐Plough **CoI:** Dr Colombel reports receiving consulting or advisory board fees from Abbott Laboratories, ActoGeniX, AstraZeneca, Bayer‐Schering Pharma, Biogen Idec, Boehringer Ingelheim, Bristol‐Myers Squibb, Cellerix, ChemoCentryx, Centocor Ortho Biotech, Cosmo Technologies, Danone France, Elan Pharmaceuticals, Genentech, Giuliani SPA, Given Imaging, GlaxoSmithKline, Merck, Millennium Pharmaceuticals, Neovacs, Ocera Therapeutics, Otsuka America Pharmaceuticals, PDL Biopharma, Pfizer, RiboVacs Biotech, Schering‐Plough, Shire Pharmaceutical, Synta Pharmaceutical, Teva Pharmaceuticals, Therakos, UCB Pharma, and Wyeth, lecture fees from Abbott Laboratories, Centocor Ortho Biotech, Elan, Falk Pharma, Ferring Pharmaceuticals, Given Imaging, Otsuka America Pharmaceuticals, PDL Biopharma, Schering‐Plough, Shire Pharmaceuticals, and UCB Pharma, grant support from Abbott Laboratories, Centocor Ortho Biotech, SyntaPharma, Otsuka America Pharmaceuticals, Bristol‐Myers Squibb, PDL Biopharma, Chiltem, AstraZeneca, Pfizer, Teva Pharmaceuticals, Lesaffre, Giuliani SPA, Danisco, Ocera Therapeutics, Danone France, Roquette, Mapi Naxis, Dysphar, Ferring Pharmaceuticals, Schering‐Plough, and UCB Pharma, and having an equity interest in Intestinal Biotech Development; Dr Sandborn, receiving consulting or advisory board fees from Abbott Laboratories (fees paid to the Mayo Clinic), ActoGeniX, AGI Therapeutics, Alba Therapeutics, Albireo, AM‐Pharma, Amgen, Ardea Biosciences, Aspreva Pharmaceuticals, Astellas Pharma, Athersys, Atlantic Healthcare Limited, Axcan Pharma, BioBalance, Bristol‐Myers Squibb, Celgene, Celek Pharmaceuticals, Cellerix, Centocor Ortho Biotech (fees paid to the Mayo Clinic), Cerimon Pharmaceutical, ChemoCentryx, CombinatoRx, CoMentis, Cosmo Technologies, Cytokine Pharmasciences, Eagle Pharmaceuticals, Eisai Medical Research, Elan Pharmaceuticals, Enteromedics, Enzo Therapeutics, Ferring Pharmaceuticals, Flexion Therapeutics, Funxional Therapeutics, Genentech, Genzyme, Given Imaging, GlaxoSmithKline, HumanGenome Sciences, Hutchison Medipharma, Ironwood Pharmaceuticals, KaloBios Pharmaceuticals, Merck Research Laboratories, MerckSerono, Millennium Pharmaceuticals, Nisshin Kyorin Pharmaceutical, Novo Nordisk, Ocera Therapeutics, Pfizer, Procter & Gamble (fees paid to the Mayo Clinic), Prometheus Laboratories, Purgenesis Technologies, Salient Pharmaceuticals, Salix Pharmaceuticals, Santarus, Schering‐Plough, Shire Pharmaceuticals (fees paid to the Mayo Clinic), Sigmoid Pharma, Sirtris Pharmaceuticals, S.L.A. Pharma, Teva Pharmaceuticals, Tillotts Pharma, Tioga Pharmaceuticals, UCB Pharma (fees paid to the Mayo Clinic), Vascular Biogenics, Ventech, Viamet Pharmaceuticals, and Wyeth, and research support from Abbott Laboratories, Bristol‐Myers Squibb, Celltech, Centocor Ortho Biotech, Genentech, Millennium Pharmaceuticals, Novartis, Otsuka America Pharmaceuticals, PDL Biopharma, Pfizer, Procter & Gamble, Robarts Research Institute, Shire Pharmaceuticals, and UCB Pharma; Dr Reinisch, receiving consulting or advisory board fees from Abbott Laboratories, Centocor Ortho Biotech, Schering‐Plough, and Genentech and lecture fees from Abbott Laboratories, Otsuka America Pharmaceuticals, and Schering‐Plough; Dr Mantzaris, receiving advisory board fees from Centocor Ortho Biotech, Schering‐Plough, Abbott Immunology, Schering‐Plough Hellas, and Abbott Hellas and lecture fees from Ferring International, Schering‐Plough Hellas, and Abbott Hellas; Dr Kornbluth, receiving consulting or advisory board fees from Abbott Laboratories, Elan‐Biogen, Centocor Ortho Biotech, and UCB Pharma, lecture fees from Abbott Laboratories and UCB Pharma, and grant support from Centocor Ortho Biotech and Abbott Laboratories; Dr Lichtiger, receiving consulting or advisory board fees from Abbott Laboratories, Centocor Ortho Biotech, Shire Pharmaceuticals, Prometheus Laboratories, and UCB Pharma, lecture fees from Abbott Laboratories, Procter & Gamble, Prometheus Laboratories, and Shire Pharmaceuticals, and grant support from Abbott Laboratories, Bristol‐Myers Pharmaceuticals, Celgene, Centocor Ortho Biotech, Osiris Pharmaceuticals, Procter & Gamble, and UCB Pharma; Dr D'Haens, receiving consulting fees from Centocor Ortho Biotech, Schering‐Plough, UCB Pharma, and Abbott and lecture fees from Schering‐Plough, Abbott Laboratories, and UCB Pharma; Drs Diamond, Broussard and Tang, being employed by Centocor Ortho Biotech and having an equity interest in Johnson & Johnson; Dr van der Woude, receiving consulting or advisory board fees from Schering‐Plough and Abbott Laboratories, lecture fees from Ferring, Tramedico, Schering‐Plough, and Abbott, and grant support from Ely Broad Foundation, Erasmus Medical Center, Schering‐Plough, and Abbott Laboratories; and Dr Rutgeerts, receiving consulting or advisory board fees from Centocor Ortho Biotech, Schering‐Plough, UCB Pharma, Abbott, Millennium, Genetech, NovImmune, ChemoCentryx, Glaxo‐SmithKline, and Italifarmako, lecture fees from Centocor Ortho Biotech, Schering‐Plough, UCB Pharma, and Abbott, and re‐search support from Centocor Ortho Biotech, Schering‐Plough, UCB Pharma, and Abbott. No other potential conflict of interest relevant to this article was reported.	
***Risk of bias***		
**Bias**	**Authors' judgement**	**Support for judgement**
Random sequence generation (selection bias)	Low risk	Quote: "Randomization was performed centrally with the use of an adaptive randomization procedure stratified according to center, the duration of Crohn's disease (<3 years or ≥3 years), and status with respect to the systemic corticosteroid dose (the equivalent of <20 mg or ≥20 mg of prednisone daily)."
Allocation concealment (selection bias)	Low risk	Central allocation concealment.
Blinding of participants and personnel (performance bias) All outcomes	Low risk	Quote: "followed through week 30 … with blinding maintained" and each group received infusions (placebo or infliximab) and tablets (placebo or azathioprine). However, there is no clarity on how this blinding was performed or if the interventions and placebo were matched and similar.
Blinding of outcome assessment (detection bias) All outcomes	Low risk	Quote: "All colonoscopies were videotaped with the use of a standard protocol and interpreted by a single reviewer, who was unaware of study‐group assignments and the timing of the procedure (i.e., at baseline or week 26)." However, it is not stated whether those collecting CDAI scores were also unaware of the assignment groups.
Incomplete outcome data (attrition bias) All outcomes	Low risk	There are relatively higher rates of attrition with AZA only having 86/162 participants (53.1%), IFX only having 111/166 participants (66.9%) and combination therapy group having 121/180 participants (67.2%). Difference was because of the adverse events.
Selective reporting (reporting bias)	Low risk	Outcomes reported match the trial registration, and appropriate for the review (EUCTR2004‐002815‐10‐GB).
Other bias	Low risk	Baseline characteristics reported and balanced. No other sources of bias apparent.

**Table:** D'Haens 1999

***Study characteristics***		
Methods	**Study design:** 4‐arm parallel RCT **Number of centres:** 4 European centres (Leuven, Belgium; Amsterdam and Leiden, the Netherlands; and Leeds, UK) **Countries:** multiple **Study chronology:** 21 June 1995 to 12 March 1996 **Setting:** NR	
Participants	**Inclusion criteria** CD diagnosis > 6 months CDAI scores 220–400 Receiving any of the following: mesalamine for ≥ 8 weeks, with the dose remaining stable during the 4 weeks before screening; maximum of 40 mg of corticosteroids per day ≥ 8 weeks, with the dose remaining stable during the 2 weeks before screening; and 6‐MP or AZA for ≥ 6 months, with the dose remaining stable during the 8 weeks before screening **Exclusion criteria** Received treatment with ciclosporin, methotrexate or experimental agents within 2 months before screening Had symptomatic stenosis or ileal strictures; proctocolectomy or total colectomy; stoma; history of allergy to murine proteins; prior treatment with murine, chimeric or humanised monoclonal antibodies; or treatment with parenteral corticosteroids or corticotropin within 4 weeks before screening **Baseline disease characteristics** Fistulating disease: NR Location of disease (ileal, colonic, etc.): ileal: 6 participants, ileocolonic: 12 participants, colonic: 12 participants Duration or length of disease since diagnosis (years): NR **Active disease characteristics** Disease activity score CDAI: IG1: 314.4 (SD 18.3) IG2: 336.8 (SD 22.1) IG3: 300.9 (SD 20.3) CG: 276 (SD 33.3) Length of active disease: NR Endoscopic disease activity scoring CDEIS: IG1: 15.1 (SD 6.9) IG2: 10.6 (SD 7.8) IG3: 13.3 (SD 6.9) CG: 8.4 (SD 6.3) **Age at beginning of study** IG1: 30.1 (SD 5.0) IG2: 30.7 (SD 8.7) IG3: 33.1 (SD 7.8) CG: 34.4 (SD 9.8) **Sex (male/female)** IG1: male = 3, female = 4 IG2: male = 3, female = 4 IG3: male = 3, female = 5 CG: male = 3, female = 5 **Smoking:** NR	
Interventions	**IG1:** single dose infliximab 5 mg/kg (IV infusion) (n = 7) **IG2:** single dose infliximab 10 mg/kg (IV infusion) (n = 7) **IG3:** single dose infliximab 20 mg/kg (IV infusion) (n = 8) **CG:** single dose of placebo (IV infusion) (n = 8) **Duration of study:** NR **Measurement time points during study:** baseline, week 4	
Outcomes	Changes in endoscopic score Changes in histological score	
Notes	**Funding:** supported in part by Centocor, Inc, Malvern, Pennsylvania, USA **CoI:** NR	
***Risk of bias***		
**Bias**	**Authors' judgement**	**Support for judgement**
Random sequence generation (selection bias)	Low risk	Randomisation performed centrally by an independent organisation (PPD Pharmaco, Austin, Texas).
Allocation concealment (selection bias)	Low risk	The IFX and placebo solutions were prepared by a pharmacist at each site. The investigators, all study personnel, and the participants were blinded to the treatment assignments.
Blinding of participants and personnel (performance bias) All outcomes	Low risk	The placebo preparation contained 0.1% human serum albumin instead of IFX and was identical in appearance to the IFX solution.
Blinding of outcome assessment (detection bias) All outcomes	Low risk	All biopsy specimens were routinely processed by haematoxylin and eosin staining and interpreted by a single, blinded gastrointestinal pathologist in random order. It is not mentioned how the CDAI was assessed.
Incomplete outcome data (attrition bias) All outcomes	Low risk	Low and balanced attrition.
Selective reporting (reporting bias)	Unclear risk	It is not entirely clear from the methods section but the intended outcomes seem to have been mucosal healing and histological changes and they were reported. However, there was no trial registration for this study.
Other bias	Low risk	No major imbalances. No other sources of bias.

**Table:** D'Haens 2008

***Study characteristics***		
Methods	**Study design:** 2‐arm parallel RCT **Number of centres:** 18 centres in Europe **Countries:** multiple **Study chronology:** May 2001 and January 2004 **Setting:** NR	
Participants	**Inclusion criteria** Aged 16–75 years CD diagnosis within past 4 years Had not previously received corticosteroids, antimetabolites or biological agents **Exclusion criteria** People who had an immediate need for surgery Symptomatic stenosis or ileal or colonic strictures with prestenotic dilation Signs, symptoms or laboratory tests that indicated severe comorbidity Documented chronic infection Positive stool culture for pathogens Positive tuberculin test or a chest radiograph consistent with tuberculosis Malignancy People who were allergic to murine proteins, pregnant, or a substance abuser **Baseline disease characteristics** Fistulating disease: NR Location of disease (ileal, colonic, etc.) – number of participants (%): Small bowel: early combined immunosuppression IG1 (n = 65): 14 (21.5%), CG: conventional management (n = 64): 15 (23.4%) Ileocolonic: IG1: early combined immunosuppression (n = 65): 31 (47.7%), CG: conventional management (n = 64): 28 (43.8%) Colonic: IG1: early combined immunosuppression (n = 65): 20 (30.8%), CG: conventional management (n = 64): 21 (32.8%) Duration or length of disease since diagnosis (years): Early combined immunosuppression: IG1: 2.0 (range 1.0–5.0) Conventional management: CG: 2.5 (range 1.0–11.0) **Active disease characteristics** Disease activity score: CDAI: early combined immunosuppression: IG1: 330 (92) Conventional management: CG: 306 (SD 80) IBDQ: early combined immunosuppression IG1: 122 (SD 33) Conventional management CG: 136 (SD 28) Length of active disease: NR Endoscopic disease activity scoring: NR **Age at beginning of study** Early combined immunosuppression: IG1: 30.0 (SD 11.8) Conventional management: CG: 28.7 (SD 10.9) **Sex (male/female)** Early combined immunosuppression: IG1: female 43 (66.2%) Conventional management: CG: female 37 (57.8%) **Smoking** Current: early combined immunosuppression: IG1: 28 (43.1%); CG: conventional management 23 (35.9%) Former: early combined immunosuppression: IG1: 8 (12.3%); CG: conventional management 16 (25.0%) Never: early combined immunosuppression; IG1: 29 (44.6%); CG: conventional management 25 (39.1%)	
Interventions	**IG1:** 67 participants; single dose IFX 5 mg/kg (IV infusion) at weeks 0, 2 and 6 with AZA 2–2.5 mg/kg per day **CG:** 64 participants; corticosteroids, AZA. IFX 5 mg/kg (in week 16) at weeks 0, 2 and 6 **Duration of study:** 2 years **Measurement time points during study** By week 14, a greater proportion of participants in the combined immunosuppression group were in remission than were participants given conventional treatment After this time point, all participants in the conventional group on AZA received IFX as well (early combined vs late) After 26 weeks, 99/65 (60.0%) participants given combined immunosuppression IG1 were in remission, compared with 23/64 (35.9%) participants given CG: controls (P = 0.0062), an absolute difference of 24.1% (95% CI 7.3 to 40.8) At 52 weeks, 40/65 (61.5%) in the early combined immunosuppression group IG1 = were in remission compared with 27/64 (42.2%) of those assigned to conventional management CG, an absolute difference of 19.4% (95% CI 2.4 to 36.3; P = 0.0278) After week 52, the proportion of participants in remission did not differ between the 2 groups. The median time to relapse after successful induction therapy at week 14 was longer for participants assigned to early immunosuppression (329.0 days, IQR 91.0–not reached) than for controls (174.5 days, IQR 780.5–274.0; P = 0.031) At week 104, there were no ulcers for 19/26 (73.1%) participants assigned to the combined immunosuppression group IG1, compared with CG: 7/23 (30.4%) of controls (P = 0.0028). The corresponding endoscopy scores were 0.7 (SD 1.5) and 3.1 (SD 2.9) (P ≤ 0.001). **Follow‐up:** NR	
Outcomes	Proportion of participants who were in remission (CDAI < 150 and no corticosteroid therapy) at week 14 Proportion given IFX, methylprednisolone and antimetabolites at any time during the study Proportion without ulcers after 24 months of treatment; and the daily dose of methylprednisolone No important differences in the occurrence of adverse events between the 2 groups. Combined immunosuppression was also more effective for both mucosal healing and serum C‐reactive protein concentration. IFX every 8 weeks could potentially have greater effects but was not yet standard practice when they initiated our trial. Although remission was more rapid for participants assigned to the early combined immunosuppression strategy than for those given conventional treatment, simultaneous initiation of antimetabolites and corticosteroids could potentially have produced similar results. Both AZA and methotrexate had a slower onset of action than IFX. Conventional management regimen reflected current clinical practice in that combined antimetabolites and corticosteroids are not commonly used as initial treatments and are not recommended by experts.	
Notes	**Funding source:** financial support for data monitoring (DRC, Wetteren, Belgium) was provided by Centocor BV and Schering Plough, who also provided IFX. Robarts Clinical Trials analysed the data (Robarts Research Institute, University of Western Ontario, London, Ontario, Canada). All authors had access to the data and jointly decided to submit the manuscript. **CoI:** PC, PV, HT, LS, AD, FVDM, JCC, PVH, GL, and FM reported no CoI. GD'H, FB, and GVA have served as consultants and speakers for Centocor and Schering Plough. SVD has acted as a consultant for Centocor. SV has received grant and research support from UCB, consultancy fees from AstraZeneca and Ferring, and speakers' fees from Schering Plough, Ferring, and UCB, and has been on the Advisory Committee for Shire, Ferring, and UCB. TO has received honoraria, consultancies, and educational grants from Centocor, Schering Plough, Essex‐Germany, UCB, and Abbott, and payments for consultancies from Shire and Boston Scientific. PR has received consulting fees, lecture fees, and grant support from Centocor and Schering Plough, and has served as an expert witness for those companies. DH has received consulting fees from Abbott, Centocor, UCB, and Schering Plough; lecture fees from Centocor, AGA and Schering Plough; and grants from Schering Plough, Abbott and UCB; and is a member of the Initiative on Crohn's and Colitis, Independent Dutch Academic Non‐profit Organisation for IBD Research. MDV has received consulting fees from Schering Plough; Altana lecture fees from Schering Plough and UCB; and grant support from AstraZeneca, Roche, Schering Plough, Novartis Fund for Scientific Research Flanders, and Special Research Fund University Ghent. SV has received consulting fees from Shire; lecture fees from Ferring, UCB, Abbott, Schering Plough, and Tillotts; and grant support from UCB. JVDW has received consulting fees from UCB Schering Plough, Elan, and Abbott; lecture fees from Schering Plough and Tramedico; and grant support from Initiative on Crohn's and Colitis. AAVBis a member of the Initiative on Crohn's and Colitis, to which Schering Plough BV and other companies that provide anti‐TNF monoclonals (Abbott BV and UCB Pharma) provide a yearly unrestricted grant. SVD has received consulting fees from Centocor, Elan, Schering Plough, and ISIS and lecture fees from Elan. BGF has received research funding from Synta, Millennium, Schering Canada, Celltech, Centocor, Elan/Biogen, Berlex, Ortho‐Biotech, Protein Design Labs, ISIS, Santarus, Schering Plough, Celgene, UCB Pharma, Napo Pharma, BMS, Abbott, and Otsuka; and consulting and lecture fees from UCB Pharma, Schering Canada, Proctor and Gamble, Elan/Biogen, Millennium, Protein Design Labs, Berlex, AstraZeneca, Celgene, Abbott, Santarus, GeneLogic, Cerimon Pharmaceuticals, Tioga Pharmaceuticals, BMS, ISIS, Serono, Teva, Genentech, and CombinatoRx.	
***Risk of bias***		
**Bias**	**Authors' judgement**	**Support for judgement**
Random sequence generation (selection bias)	Low risk	Participants were randomised according to a "computer‐generated schedule".
Allocation concealment (selection bias)	Low risk	Person independent of the trial performed the allocation.
Blinding of participants and personnel (performance bias) All outcomes	High risk	Open‐label trial.
Blinding of outcome assessment (detection bias) All outcomes	Unclear risk	Based on authors stating, "Allocation was not concealed from investigators or patients", authors were contacted to confirm that the outcome assessors were unaware of the treatment assignments.
Incomplete outcome data (attrition bias) All outcomes	Low risk	Attrition was accounted for and balanced between both groups.
Selective reporting (reporting bias)	Low risk	Trial registration was NCT00554710 and the study reported according to the primary outcomes stated – proportion of participants with corticosteroid‐free remission and remission without surgical resection.
Other bias	Low risk	Baseline characteristics reported and balanced in both groups. No other apparent sources of bias.

**Table:** Duan 2013

***Study characteristics***		
Methods	**Study design:** 3‐arm RCT **Number of centres:** 1 **Countries:** China **Study chronology:** March 2010 to September 2012 **Setting:** secondary care	
Participants	**Inclusion criteria** People with CD who were treated at the Hunan Provincial Corps Hospital of the Chinese People's Armed Police Force from March 2010 to September 2012 Conform to the consensus opinion on the diagnosis and treatment of inflammatory bowel disease formulated by the Inflammatory Bowel Disease Collaborative Group of the Chinese Medical Association in 2007 **Exclusion criteria** Active infection (including tuberculosis, viral hepatitis or other potential infections) Nerve demyelination disease Moderate‐to‐severe congestive heart failure Malignant tumour CD complicated with intestinal stenosis Allergic to mouse‐derived protein components Age < 14 years Pregnant or breastfeeding women **Baseline disease characteristics** Fistulating disease: NR Location of disease (ileal, colonic, etc.): NR Duration or length of disease since diagnosis: NR **If active disease population** Disease activity score: NR Length of active disease: NR Endoscopic disease activity scoring: NR **Active disease characteristics** Disease activity score: NR Length of active disease: NR Endoscopic disease activity scoring: NR **Age at beginning of study** **IG1:** average (not specified whether mean, median, or mode) age of 34.4 ± 12.6 years (value after '±' not specified whether SD or range) **IG2:** average (not specified whether mean, median, or mode) age of 35.4 ± 18.8 years (value after '±' not specified whether SD or range) **CG:** average (not specified whether mean, median or mode) age of 36.2 ± 17.6 years (value after '±' not specified whether SD or range) **Sex (male/female)** **IG1:** males 7 (87.5%), females 1 (12.5%) **IG2:** males 8 (100%), females 0 (0%) **CG:** males 6 (75%), females 2 (25%) **Smoking:** NR	
Interventions	**IG1:** IFX 5 mg/kg at weeks 0, 2 and 6 to induce remission followed by IFX 5 mg/kg every 8 weeks to maintain remission **IG2:** combination treatment group; AZA 2.5 mg/kg qd, and on the 0th and 2nd week, IFX 5 mg/kg was given for 6 weeks, and then IFX 5 mg/kg was given every 8 weeks **CG:** AZA 2.5 mg/kg qd **Duration of study:** 26 weeks **Measurement time points during study:** week 26 **Follow‐up:** NR	
Outcomes	CDAI assessment and endoscopic examination performed at week 26 to evaluate treatment efficacy Adverse effects during the course of treatment	
Notes	**Funding source:** NR **CoI:** NR	
***Risk of bias***		
**Bias**	**Authors' judgement**	**Support for judgement**
Random sequence generation (selection bias)	Unclear risk	Quote: "patients were randomly divided into infliximab group, azathioprine group, and infliximab combined with azathioprine group", but do not clarify how this was performed. Contacted study authors but received no response for clarification on unclear risk of bias items.
Allocation concealment (selection bias)	Unclear risk	Study authors did not describe any method of concealment during assignment to treatment groups.
Blinding of participants and personnel (performance bias) All outcomes	Unclear risk	Study authors did not describe any evidence of blinding participants or personnel.
Blinding of outcome assessment (detection bias) All outcomes	Unclear risk	No evidence or method of blinding (or both) the endoscopist or clinician collecting CDAI score or interpreter was described.
Incomplete outcome data (attrition bias) All outcomes	Low risk	No evidence of attrition from the original 24 enroled and randomised to each group.
Selective reporting (reporting bias)	Unclear risk	No protocol or trial registration found, but study reports on CDAI and endoscopic mucosal healing
Other bias	Low risk	Reported baseline characteristics of no significant difference – age, gender. No other sources are apparent.

**Table:** Hanauer 2002

***Study characteristics***		
Methods	**Study design:** 3‐arm RCT **Number of centres:** 55 sites **Countries:** many (in North America, Europe and Israel) **Study chronology:** 26 February 1999 to 24 January 2000 **Setting:** secondary care and educational institutions	
Participants	Only the non‐responders population was relevant for this review. **Inclusion criteria** People with CD of ≥ 3 months' duration with a CDAI‐17 score 220–400 Receiving 5‐aminosalicylates or antibiotics (if the dose remained constant for 4 weeks before the screening visit); corticosteroids (prednisone, prednisolone or budesonide) at the equivalent of ≤ 40 mg/day of prednisone (stable dose for 3 weeks); azathioprine and 6‐MP (stable dose for 8 weeks) or methotrexate (stable dose for 6 weeks). Participants not receiving medical therapy had to have discontinued treatment for ≥ 4 weeks before screening. **Exclusion criteria** Received previous treatment with IFX or any other agent targeted at TNF. Non‐responders were excluded from the assessment of the co‐primary endpoints **Baseline disease characteristics** Fistulating disease: NR Location of disease (ileal, colonic, etc.) NR per group. For week‐2 non‐responders: ileum 63/237 (27%), colon 35/237 (15%), ileum and colon 139/237 (59%), gastroduodenum 19/238 (7%) Duration or length of disease since diagnosis NR per group. Study authors were not clear whether the disease duration reported in their paper was duration of disease since diagnosis or duration of active disease but given the long times, it was likely to be duration since diagnosis. For week 2, non‐responders: median 9.3 (range 4.6–15.3) **Active disease characteristics** **Disease activity score (CDAI)** NR per group. For week 2, non‐responders: median 291 (IQR 249–340) **Length of active disease:** NR **Endoscopic disease activity scoring:** NR **Age (years) at beginning of study** For week 2, non‐responders: 37 (range 30–46) **Sex (male/female)** NR per group. For week 2, non‐responders: male 109 (46%), female 129 (54%) **Smoking:** NR	
Interventions	**IG1:** IFX infusion, each infusion 5 mg/kg at week 2 (start point of maintenance study), week 6 and every 8 weeks thereafter until week 46 **IG2:** IFX infusion, 5 mg/kg infusions at weeks 2 and 6. IFX 10 mg/kg every 8 weeks thereafter until week 46 **CG:** placebo; identical in appearance to IFX infusion. Infusions at week 2, week 6, and every 8 weeks thereafter until week 46 **Duration of study:** 52 weeks (weeks 2–54) as first 2 weeks are non‐randomised induction period **Measurement time points during study:** participants were assessed at weeks 0, 2, 6, 10, 14, 22, 30, 38, 46 and 54 **Follow‐up measurements after study end:** NR	
Outcomes	**Primary outcomes as defined by study authors:** the prespecified co‐primary efficacy endpoints were the proportion of week‐2 responders in clinical remission at week 30, and the time to loss of response up to week 54 amongst week‐2 responders. Later, in an amendment made to the original protocol, the proportion of week‐2 responders who were in remission at week 30, as defined by a CDAI score < 150 points, was added as a co‐primary efficacy endpoint to provide an earlier assessment of the efficacy of maintenance IFX infusions. **Secondary outcomes as defined by study authors:** secondary objectives included the assessment IFX's corticosteroid‐sparing effects and safety in numerous participants.	
Notes	**Funding source:** role of the funding source: this study was designed by a committee composed of Centocor staff members and the ACCENT Steering Committee members. Centocor staff collected data from all clinical sites to create the clinical database. Centocor staff members and members of the ACCENT Steering Committee analysed and interpreted the data, wrote the paper, and agreed to submit it for publication. The principal investigators approved the content of the paper before submission. **CoI:** SB Hanauer has acted as a consultant for, received honoraria from, provided paid expert testimony for, and received travel grants from Centocor. BG Feagan has received honoraria from Centocor. GR Lichtenstein has acted as a consultant for, received honoraria from, and received travel grants from Centocor. LF Mayer has acted as a consultant for, and received honoraria from Centocor. DC Wolf has acted as a consultant for, received honoraria from, and received travel grants from Centocor. A Olson and W Bao are employees of Centocor. P Rutgeerts has provided paid expert testimony for Centocor.	
***Risk of bias***		
**Bias**	**Authors' judgement**	**Support for judgement**
Random sequence generation (selection bias)	Low risk	Study authors mention random assignment via adaptive randomisation voice response system.
Allocation concealment (selection bias)	Low risk	Quotes: "allocation of patients to a treatment group was done with an adaptive stratified design" and "allocate patients centrally to treatment based on the current balance of treatment groups within each stratum".
Blinding of participants and personnel (performance bias) All outcomes	Low risk	Quotes: "Neither the patients nor study investigators were aware of the treatment assignment" and "pharmacist prepared the infusion (infliximab [Remicade]) or an identically appearing placebo".
Blinding of outcome assessment (detection bias) All outcomes	Low risk	Quotes: "Neither the patients nor study investigators were aware of the treatment assignment" and "pharmacist prepared the infusion (infliximab [Remicade]) or an identically appearing placebo".
Incomplete outcome data (attrition bias) All outcomes	Unclear risk	This group was only followed up for safety. Safety data were presented for the entire cohort and not separately throughout the end of the study.
Selective reporting (reporting bias)	Unclear risk	Safety data and primary efficacy data were given for the entire cohort and not separately. The trial registration information was very unclear.
Other bias	Unclear risk	Baseline characteristics reported were summarised for all participants, responders and non‐responders. Authors were asked to confirm that the baseline characteristics of the 3 treatment groups were balanced within the responder and non‐responder groups, and not just between them.

**Table:** Lemann 2006

***Study characteristics***		
Methods	**Study design:** 2‐arm RCT **Number of centres:** 22 sites **Countries:** France **Study chronology:** recruitment of participants took place from June 2000 to May 2002 **Setting:** all physicians were members of the Groupe d'Etude Thérapeutique des Affections Inflammatoires du tube Digestif (GETAID)	
Participants	**Inclusion criteria** Aged ≥ 18 years with luminal steroid‐dependant CD. Steroid dependency defined as: prednisone for ≥ 6 months at ≥ 10 mg/day with no interruption for > 2 months within last 6 months ≥ 2 clinical luminal relapses with attempted tapering of steroids, leading to dose increase >10 mg/day last attempt of steroid tapering had to be within past 6 months. **Exclusion criteria** Contraindication to AZA/6‐MP or to IFX according to labelling recommendations Treatment with an immunosuppressive drug other than AZA/6‐MP in the past 6 months Previous use of IFX or other antitumour necrosis factor drugs including thalidomide Concomitant treatment with aminosalicylates, budesonide, topical steroids or artificial nutrition Presence of ≥ 1 of the following conditions: symptomatic stricture, intra‐abdominal abscess or infection, severe sepsis within the past 3 months, tuberculosis (because the bacillus Calmette‐Guerin vaccination still is recommended in France, participants with a tuberculin skin test > 10 mm and a bacillus Calmette‐Guerin vaccination performed > 10 years before the tuberculin skin test were excluded), history of B or C hepatitis, HIV infection, liver failure, pregnancy, breastfeeding or participation in pharmaceutical research within the past 3 months **Baseline disease characteristics** Fistulating disease: NR Location of disease: Ileal: *Failure stratum:* IG: n = 5 (20%), CG: n = 3 (11%) *Naive stratum:* IG: n = 11 (35%), CG: n = 4 (15%) Colon: *Failure stratum:* IG: n = 8 (32%), CG: n = 14 (50%) *Naive stratum:* IG: n = 4 (13%), CG: n = 7 (26%) Ileocolonic: *Failure stratum:* IG: n = 12 (48%), CG: n = 11 (39%) *Naive stratum:* IG: n = 16 (52%), CG: n = 16 (59%) Active perianal disease: *Failure stratum:* IG: n = 4 (14%), CG: n = 10 (35%) *Naive stratum:* IG: n = 10 (32%), CG: n = 2 (7%) Duration or length of disease since diagnosis (median (years)) *Failure stratum:* IG: 5 (IQR 4–10), CG: 7 (IQR 3–11) *Naive stratum:* IG: 3 (IQR 1–6), CG: 4 (IQR 1–8) **Active disease characteristics** Disease activity score: CDAI: median *Failure stratum:* IG: 240 (IQR 219–281), CG: 181 (IQR 154–259) *Naive stratum:* IG: 146 (IQR 90), CG: 112 (IQR 42–262) Length of active disease: median disease duration (years): NR Endoscopic disease activity scoring: CDEIS (n = 52): median *Failure stratum:* IG: 9 (IQR 4–14), CG: 9 (IQR 6–15) *Naive stratum:* IG: 11 (IQR 6–16), CG: 6 (IQR 3–14) **Age at beginning of study** (**median)** *Failure stratum:* IG: 26 (range 22–37), CG: 29 (range 23–33) *Naive stratum:* IG: 27 (range 22–38), CG: 26 (range 22–36) **Sex (male/female)** Female n: *Failure stratum:* IG: 18 (69%), CG: 20 (69%) *Naive stratum:* IG: 12 (39%), CG: 12 (43%) Male n: *Failure stratum:* IG: 8, CG: 9 *Naive stratum:* IG: 15, CG: 19 **Smoking:** NR	
Interventions	**IG:** IFX 5 mg/kg, IV infusion over 2 hours **CG:** placebo, IV infusion over 2 hours All participants were treated with AZA 2–3 mg/kg per day or 6‐MP 1–1.5 mg/kg per day. Participants previously treated with AZA or 6‐MP (failure stratum) continued their treatment at the same dose; in the naive‐stratum participants, AZA 2–2.5 mg/kg per day was started 1 week after the first IFX infusion (to differentiate adverse effects related to IFX from those related to AZA). The AZA or 6‐MP dose had to be maintained at a stable dose throughout the study, except for participants who experienced toxicity related to the drug. **Duration of study:** 24 weeks **Measurement time points during study:** weeks 0, 2, 6, 12 and 24 **Follow‐up measurements after study end:** follow‐up at week 52	
Outcomes	**Primary outcomes as defined by study authors** Rate of success, defined as a clinical remission (CDAI 150) off steroids at week 24 **Secondary outcomes as defined by study authors** Success rate at week 12 Rate of steroid resistance Cumulative dose of prednisone at week 24 Steroids adverse effect score at weeks 6, 12 and 24 Endoscopic improvement between inclusion and week 24 Adverse events	
Notes	**Funding source:** supported by Groupe d'Etude Thérapeutique des Affections Inflammatoires du tube Digestif (GETAID), and by grants from Schering Plough, France, with the specific help of Gérard Trape and Yves‐Dominique Henry. Drugs were provided by Schering Plough, France. All data analysis and manuscript writing were performed independently by the GETAID, without the involvement of representatives of Schering Plough. **CoI:** NR	
***Risk of bias***		
**Bias**	**Authors' judgement**	**Support for judgement**
Random sequence generation (selection bias)	Low risk	Quote: "randomization was performed centrally, using permutation tables". Contacted author who confirmed that the permutation tables used were provided in J Lellouch and P Lazar, Méthodes statistiques en expérimentation biologique. Flammarion Médecine Sciences 1974, pp 259 and 258.
Allocation concealment (selection bias)	Low risk	Authors stated that randomisation was performed centrally suggesting concealment. Author clarification: "When an investigator wanted to include a new patient and to obtain the treatment to be given to this patient, the investigator sent me by fax in my lab a randomization request sheet including patient's identification (3 first letters last name, 2 first letters first name), center, stratum, and the major inclusion criteria. If all inclusion criteria were satisfied, I updated the first free line of the list of randomization of the stratum within the center with patient's identification, investigator's name, date of randomization, the first free treatment number in the list of treatment numbers corresponding to assigned treatment through randomization. The list of treatment numbers per treatment was common to my center and to Shering delivery service. I completed the randomization request sheet with patient number within the stratum and center, treatment number and sent it to investigator's center. I sent in parallel to Shering delivery service the same data plus treatment assigned, placebo or Remicade, allowing this service to check coherence with treatment number and name. Shering delivery service sent to the pharmacy of the investigator's hospital the blind treatment with patients' identification and treatment number. If at least one inclusion criteria was missing or not satisfied, I sent back to the investigator the randomization request sheet explaining why treatment allocation to his/her patient could not be performed. Thus, I had no contact with patients, I had no contact with investigators except through the randomization request sheet, I had knowledge of the treatment of a new patient when looking at the randomization list of the stratum and center after reception of the randomization request sheet."
Blinding of participants and personnel (performance bias) All outcomes	Low risk	Quotes: "Neither the patients nor the study investigators were aware of the treatment assigned" and that "identical placebo" to IFX (Remicade) was administered.
Blinding of outcome assessment (detection bias) All outcomes	Low risk	Contacted study authors and confirmed that the outcome assessors were not aware of participant's treatment.
Incomplete outcome data (attrition bias) All outcomes	Low risk	Attrition was accounted for and balanced in both groups. Reasons for attrition were provided adequately and did not affect outcomes.
Selective reporting (reporting bias)	Unclear risk	No trial registration or published protocol found. Authors report primary outcome as stated in their method section – clinical remission with CDAI score < 150 off steroids at week 24.
Other bias	Low risk	Baseline characteristics have been reported and are overall balanced in both treatment groups. No other sources of bias apparent.

**Table:** Present 1999

***Study characteristics***		
Methods	**Study designs:** 3‐arm RCT **Number of centres:** 12 sites **Countries:** multiple **Study chronology:** 30 May to 1 October 1996. Recruitment study completed February 1998 **Setting:** NR	
Participants	**Inclusion criteria** Aged 18–65 years and who had single or multiple draining abdominal or perianal fistulas of ≥ 3 months' duration as a complication of CD that had been confirmed by radiography, endoscopy or pathological examination. Participants could receive concomitant therapy. Acceptable regimens were aminosalicylates at a dosage that had been stable for > 4 weeks before screening; oral corticosteroids at ≤ 40 mg/day that had been stable for > 3 weeks; methotrexate given for ≥ 3 months at a dosage that had been stable for > 4 weeks; AZA or 6‐MP gave for ≥ 6 months at a dosage that had been stable for > 8 weeks; and antibiotics at a dosage that had been stable for > 4 weeks. If participants were not currently receiving treatment with any of these medications, they had to have discontinued therapy ≥ 4 weeks before enrolment Men and women with reproductive potential were required to use an acceptable form of contraception throughout the study and for 6 months after the final infusion **Exclusion criteria** People treated concurrently with ciclosporin Treatment with investigational agents or the use of any medication to reduce the concentration of TNF‐α was not allowed within 3 months before enrolment Other complications of CD, such as current strictures or abscesses; presence of a stoma created < 6 months before enrolment History of allergy to murine proteins Previous treatment with IFX **Baseline disease characteristics** Number of fistulae CG: < 1: 13 (42); > 1: 18 (58) IG1: < 1: 15 (48); > 1: 16 (52) IG2: < 1: 14 (44); > 1: 18 (56) Location of disease (ileal, colonic, etc.) CG: ileum 3 (10%), colon 9 (29%), ileum and colon 19 (61%) IG1: ileum 7 (23%), colon 7 (23%), ileum and colon 17 (55%) IG2: ileum 14 (15%), colon 26 (28%), ileum and colon 54 (57%) Duration or length of the disease since diagnosis CG: 12.0 (SD 7.9) IG1: 13.6 (SD 9.5) IG2: 11.5 (SD 8.2) **Active disease characteristics** Disease activity score: CG: CDAI 192.9 (SD 92.0) IG1: CDAI 184.4 (SD 98.5) IG2: CDAI 184.9 (SD 97.5) Endoscopic disease activity scoring: NR **Age at beginning of study:** median age (years) CG: 35.4 (SD 8.6) IG1: 41.2 (SD 12.2) IG2: 5.0 (SD 12.3) **Sex (male/female)** CG: male: 17 (55%), female: 14 (45%) IG1: male: 15 (48%), female 16 (52%) IG2: male: 12 (38%), female 20 (62%) **Smoking:** NR	
Interventions	**CG:** placebo **IG1:** IFX 5 mg/kg **IG2:** IFX 10 mg/kg **Duration of study:** NR **Measurement time points during the study** Clinical and laboratory assessments at weeks 2, 6, 10, 14 and 18. Blood samples were drawn at each study visit and at weeks 26 and 34 to determine the serum concentration of IFX **Follow‐up measurements after the study end:** NR	
Outcomes	**Primary outcomes as defined by study authors** Reduction of ≥ 50% from baseline in the number of draining fistulas observed at ≥ 2 consecutive study visits **Secondary outcomes as defined by study authors** Closure of all fistulas	
Notes	**CoI:** Dr Hanauer, Dr Podolsky and Dr Sands have served as paid consultants to Centocor. Dr Present and Dr Hanauer have received honorariums from Centocor for lectures. Dr Mayer owns stock in Centocor.	
***Risk of bias***		
**Bias**	**Authors' judgement**	**Support for judgement**
Random sequence generation (selection bias)	Unclear risk	Randomisation was performed by an independent organization (PPD Pharmaco, Austin, Texas) using a stratified treatment assignment, methods not described.
Allocation concealment (selection bias)	Low risk	Central allocation by PPD Pharmaco.
Blinding of participants and personnel (performance bias) All outcomes	Low risk	Double‐blind study. Placebo was identical in appearance to the IFX solution.
Blinding of outcome assessment (detection bias) All outcomes	Unclear risk	Not mentioned.
Incomplete outcome data (attrition bias) All outcomes	Low risk	Dropouts were reported and distributed evenly across treatment groups.
Selective reporting (reporting bias)	High risk	Trial registration NCT00004941. However, outcomes were not defined and primary outcome (50% draining of fistulas) used was uncommon.
Other bias	Low risk	Study appeared free of other sources of bias.

**Table:** Sands 2004b

***Study characteristics***		
Methods	**Study design:** 2‐arm RCT **Number of centres:** 45 sites **Countries:** North America (34 sites), Europe (9 sites) and Israel (2 sites). Exact countries NR **Study chronology:** 21 January 2000 to 17 October 2001 **Setting:** secondary care	
Participants	Only the non‐responders population was relevant for this review. **Inclusion criteria** Men and women aged ≥ 18 years with CD with single or multiple draining fistulas, including perianal fistulas and enterocutaneous fistulas, for ≥ 3 months. Women with rectovaginal fistulas were included if they had ≥ 1 other enterocutaneous draining fistula. Setons were permitted at screening but were required to be removed by week 2. Concurrent therapies for CD, including stable doses of 5‐aminosalicylates, oral corticosteroids, AZA, 6‐MP, mycophenolate mofetil, methotrexate and antibiotics, were permitted. Participants being extracted here were those that did not have a response (reduction of ≤ 50% from baseline in the number of draining fistulas at consecutive visits ≥ 4 weeks apart. A participant was classified as having a response if a response was observed at both weeks 10 and 14) at week 14 of the study. Responders are being extracted separately **Exclusion criteria** Person had a stricture or abscess for which surgery might be indicated or if they had previously been treated with IFX. **Baseline disease characteristics** Fistulating disease: NR per group Location of disease (ileal, colonic, etc.): NR per group Duration or length of disease since diagnosis: NR per group Disease activity score: NR per group Length of active disease: NR Endoscopic disease activity scoring: NR **Active disease characteristics** Disease activity score: NR per group Length of active disease: NR Endoscopic disease activity scoring: NR **Age at beginning of study:** NR per group **Sex (male/female):** NR per group **Smoking:** NR per group	
Interventions	**IG:** IFX 5 mg/kg at weeks 14, 22, 30, 38 and 46. Beginning at week 22, participants who had a loss of response (defined by recrudescence of draining fistulas, the need for additional therapy for persistent or worsening luminal disease activity, the need for a surgical procedure for CD, or the discontinuation of the study medication owing to a perceived lack of efficacy) were eligible to cross over to maintenance treatment with IFX 10 mg/kg **CG:** placebo at weeks 14, 22, 30, 38 and 46. Beginning at week 22, participants who had a loss of response (defined by recrudescence of draining fistulas, the need for additional therapy for persistent or worsening luminal disease activity, the need for a surgical procedure for CD, or the discontinuation of the study medication owing to a perceived lack of efficacy) were eligible to cross over to maintenance treatment with IFX 5 mg/kg **Duration of study:** 41 weeks (weeks 14–54) **Measurement time points during study:** weeks 0, 2, 6, 10, 14, 22, 30, 38, 46 and 54 **Follow‐up measurements after study end:** NR	
Outcomes	**Primary outcomes as defined by study authors** Time to loss of response amongst participants who had a response at week 14 and underwent randomisation. **Secondary outcomes as defined by study authors** Proportion of participants who had a response to continued treatment after having had no response to initial treatment at week 14	
Notes	**Funding source:** supported by Centocor. Dr Sands was supported in part by a grant (K23 DK002850) from the National Institutes of Health. **CoI:** Dr Sands reports having served as a paid consultant on advisory boards to Centocor, Elan/Biogen, Protein Design Labs, Celltech, Otsuka America Pharmaceutical, and Berlex and having received lecture fees from Centocor and AstraZeneca and grant support from Centocor, Abbott, and Elan. Dr Anderson reports having received consulting fees from Bristol‐Myers Squibb, Hoffmann–LaRoche, Agouron, and Axcan Pharma and lecture fees from Schering Canada, Hoffmann–LaRoche, and Glaxo‐Welcome. Dr Bernstein reports having received consulting fees from Elan, Abbott, and Novartis Canada, owning stock in Pfizer, and having received grant support from Ferring Canada. Dr Feagan reports having received consulting and lecture fees from Centocor and Schering‐Plough. Dr Fedorak reports having received consulting fees from Abbott, Celltech, and Serono and grant support from Centocor, Abbott, Serono, Millennium Pharmaceuticals, and Wyeth. Dr Korzenik reports having received consulting fees from Amgen, Isis Pharmaceuticals, Berlex, and Incara, lecture fees from Centocor, Procter & Gamble, and Berlex, and grant support from Rhodia. Dr Lashner reports having received lecture fees from AstraZeneca, Procter & Gamble, and Prometheus Laboratories. Dr Onken reports owning stock in Schering‐Plough. Dr Rutgeerts reports having received consulting and lecture fees from Centocor, Schering‐Plough, Celltech, Serono, and Elan/Biogen and grant support from Centocor and Schering‐Plough. Dr Wild reports having received consulting and lecture fees from Schering Canada. Dr Wolf reports having received consulting fees from Centocor, AstraZeneca, Janssen, and Otsuka America Pharmaceutical. Dr van Deventer reports having received consulting fees from Centocor, Schering‐Plough, Merix Bioscience, Elan, Biogen, and Serono, owning stock in Merix Biosciences and Amsterdam Molecular Therapeutics, having received lecture fees from Centocor, Schering‐Plough, AstraZeneca, and Elan, and having received grant support from Centocor, Protein Design Labs, Serono, and Genzyme. Mr Marsters, Dr Travers, and Dr Blank are employees of Centocor and own Johnson & Johnson stock options.	
***Risk of bias***		
**Bias**	**Authors' judgement**	**Support for judgement**
Random sequence generation (selection bias)	Low risk	A computer‐generated adaptive randomisation scheme was used, which included the study site, the number of draining fistulas at baseline (1 vs > 1), and the presence or absence of active bowel disease at baseline (active bowel disease was considered to be present if the CDAI was ≥ 150) as stratification factors.
Allocation concealment (selection bias)	Low risk	A pharmacist prepared each infusion of IFX or an identical appearing placebo. Neither the participants nor the study investigators were aware of the treatment assignment. Cross‐overs were blinded so that participants and physicians remained unaware of the treatment assignment.
Blinding of participants and personnel (performance bias) All outcomes	Low risk	A pharmacist prepared each infusion of IFX or an identical appearing placebo. Neither the participants nor the study investigators were aware of the treatment assignment. Cross‐overs were masked so that participants and physicians remained unaware of the treatment assignment.
Blinding of outcome assessment (detection bias) All outcomes	Unclear risk	Not mentioned. Contacted authors for clarification.
Incomplete outcome data (attrition bias) All outcomes	Low risk	Low and balanced attrition that did not affect outcomes.
Selective reporting (reporting bias)	High risk	The reported outcomes had shifted from those published in the trial registration.
Other bias	Low risk	Baseline characteristics are balanced across the study arms. No other sources of bias are apparent.

**Table:** Targan 1997

***Study characteristics***		
Methods	**Study design:** 4‐arm parallel RCT **Number of centres:** 18 centres in North America and Europe **Countries:** multiple **Study chronology:** 21 June 1995 to 12 March 1996 **Setting:** NR	
Participants	**Inclusion criteria** CD diagnosis > 6 months CDAI scores 220–400 Receiving any of the following: mesalamine for ≥ 8 weeks, with the dose remaining stable during the 4 weeks before screening; maximum of 40 mg of corticosteroids per day for ≥ 8 weeks, with the dose remaining stable during the 2 weeks before screening; and 6‐MP or AZA for ≥ 6 months, with the dose remaining stable during the 8 weeks before screening. **Exclusion criteria** Had received treatment with ciclosporin, methotrexate or experimental agents within 3 months before screening If they met any of the following criteria: symptomatic stenosis or ileal strictures; proctocolectomy or total colectomy; stoma; history of allergy to murine proteins; prior treatment with murine, chimeric or humanised monoclonal antibodies; or treatment with parenteral corticosteroids or corticotropin within 4 weeks before screening **Baseline disease characteristics** Fistulating disease: NR Location of disease (ileal, colonic, etc.) and number of participants (%) Ileal: IG1: 3 (11%), IG2: 4 (14%), IG3: 2 (7%), CG: 8 (32%) Ileocolonic: IG1: 15 (56%), IG2: 14 (50%), IG3: 19 (68%), CG: 10 (40%) Colonic: IG1: 9 (33%), IG2: 10 (36%), IG3: 7 (25%), CG: 7 (28%) Previous segmental resection: IG1: 12 (44%), IG2: 14 (50%), IG3: 14 (50%), CG: 13 (52%) Duration or length of disease since diagnosis (years): IG1: 12.5 (SD 10.3) IG2: 11.5 (SD 9.6) IG3: 13.5 (SD 8.8) CG: 10.4 (SD 7.7) **Active disease characteristics** Disease activity score: CDAI: IG1: 312 (SD 56), IG2: 318 (SD 59), IG3: 307 (SD 50), CG: 288 (SD 54) IBDQ: IG1: 122 (SD 29), IG2: 116 (SD 23), IG3: 118 (SD 28), CG: 128 (SD 29) Length of active disease: NR Endoscopic disease activity scoring: NR **Age at beginning of study** IG1: 37.0 (SD 11.8) IG2: 39.3 (SD 10.6) IG3: 36.0 (SD 9.7) CG: 38.5 (SD 11.0) **Sex (male/female) (numbers of participants)** IG1: male = 14/27 (52%), female = 13/27 (48%) IG2: male = 13/28 (46%), female = 15/28 (54%) IG3: male = 13/28 (46%), female = 15/28 (54%) CG: male = 15/25 (60%), female = 10/25 (40%) **Smoking:** NR	
Interventions	**IG1:** single dose cA2 at 5 mg/kg (IV infusion) (n = 27) **IG2:** single dose cA2 at 10 mg/kg (IV infusion) (n = 28) **IG3:** single dose cA2 at 20 mg/kg (IV infusion) (n = 28) **CG:** single dose of placebo (IV infusion) (n = 25) **Duration of study:** 12 weeks **Measurement time points during study:** screening (−7 days), baseline, and weeks 2, 4, 8 and 12	
Outcomes	**Primary outcome** Clinical response defined as a decrease in CDAI of ≥ 70 points at week 4, not associated with concomitant medication Remission defined as CDAI < 150 AND IBDQ score 170–190	
Notes	**Funding source:** supported by Centocor, Inc., and by a grant (FD‐R‐001276) from the Food and Drug Administration Orphan Products Development Division. **CoI:** Drs Hanauer, van Deventer, Present and Rutgeerts have received honorariums from Centocor for lectures.	
***Risk of bias***		
**Bias**	**Authors' judgement**	**Support for judgement**
Random sequence generation (selection bias)	Unclear risk	Quote: "Randomization was performed centrally by an independent organization (PPDPharmaco, Austin, Tex.)" Contacted study authors.
Allocation concealment (selection bias)	Low risk	Quote: "Randomization was performed centrally by an independent organization (PPDPharmaco, Austin, Tex.)."
Blinding of participants and personnel (performance bias) All outcomes	Low risk	Quote: "The investigators, all other study personnel, and the patients were blinded to the treatment assignments."
Blinding of outcome assessment (detection bias) All outcomes	Low risk	Quote: "The investigators, all other study personnel, and the patients were blinded to the treatment assignments."
Incomplete outcome data (attrition bias) All outcomes	Low risk	No attrition.
Selective reporting (reporting bias)	Unclear risk	Outcomes reported per the trial registration. However, the safety data included non‐randomised participants.
Other bias	Low risk	Baseline characteristics were reported for and balanced in all groups. No other apparent sources of bias.

**Table:** Ye 2019

***Study characteristics***		
Methods	**Study design:** RCT **Number of centres:** 58 sites **Countries**: 16 countries (Belgium, Brazil, Denmark, France, Germany, Hungary, Italy, Israel, Mexico, the Netherlands, Poland, Republic of Korea, Romania, Russia, Ukraine, the USA) **Study chronology:** 20 August 2014 to 15 February 2017 **Setting:** NR	
Participants	**Inclusion criteria:** NR in article **Exclusion criteria:** NR in article **Baseline disease characteristics** Fistulating disease: NR Location of disease (ileal, colonic, etc.): NR Duration or length of disease since diagnosis (years): ≥ 12 weeks **Active disease characteristics** Disease activity score: CDAI: IG1: n = 296.3 (SD 54.3) GC: n = 295.7 (SD 55.46) Length of active disease: Endoscopic disease activity scoring: SES‐CD score: IG1: n = 9.6 CG: n = 9.8 **Age at beginning of study** IG1: 35.0 years CG: 32.0 years **Sex (male/female)** IG1: male = 63, female = 48 CG: male = 60, female = 49 **Smoking** IG1: current or former smoker n = 35; non‐smoker n = 76 CG: current or former smoker n = 39; non‐smoker n = 70	
Interventions	5 mg/kg of each drug **IG:** 111 randomly assigned. 56 to CT‐P1356 to CT‐P13–CT‐P1355. 55 to CT‐P13–IFX **CG:** 109 randomly assigned. 54 to IFX–IFX. 55 to IFX–CT‐P13 **Duration of study:** 30 weeks **Measurement time points during study:** baseline, and weeks 6, 14, 30 and 54 **Follow‐up measurements after study end:** week 54	
Outcomes	**Primary outcomes as defined by study authors** CDAI‐70 response at week 6 **Secondary outcomes as defined by study authors** CDAI‐70 response at week 14 Clinical remission (defined as an absolute CDAI < 150 points) at weeks 6 and 14 SIBDQ scores at weeks 0, 6 and 14 Secondary endpoints were assessed again at weeks 30 and 54 in participants who continued the study.	
Notes	**Funding source:** Celltrion (Incheon, South Korea) and Pfizer (New York, New York, USA). Medical writing support, including development of a draft outline and subsequent drafts in consultation with the authors, assembling tables and figures, collating author comments, copyediting, fact checking, and referencing, was provided by Emma Prest, at Aspire Scientific Limited (Bollington, UK), and was funded by Celltrion. **CoI:** BDY reports personal fees and non‐financial support from Celltrion during the conduct of the study and personal fees from Abbvie Korea, Cornerstones Health, Ferring Korea, IQVIA, Janssen Korea, Kangstem Biotech, Kuhnil Pharm, Robarts Clinical Trials, Shire Korea, and Takeda Korea outside the submitted work. AL reports consultancy and lecture fees from Takeda and lecture fees from Abbvie, Celltrion, and Janssen outside the submitted work. MLS reports advisory board fees from Janssen, Mundipharma, and Pfizer and advisory board and speaker fees from Abbvie and Takeda outside the submitted work. R‐BM reports personal fees from Amgen outside the submitted work; personal fees and non‐financial support from Abbvie, Alfa Sigma, Alvogen, Dr Reddys, Egis Pharmaceutical, MSD, and Takeda outside the submitted work; and grants from Abbvie outside the submitted work. K‐ML reports consultancy and lecture fees from Takeda and consultancy fees from Celltrion outside the submitted work. CSE reports consultancy and lecture fees from Celltrion outside the submitted work. SJL is an employee of, and has stock options for, Celltrion. SYL and HUK are employees of Celltrion. SS reports consulting (advisory board) fees from AbbVie, Biogen/Samsung, Boehringer Ingelheim, Celltrion, Merck, Pfizer, Sandoz, Shire, and UCB outside the submitted work. HF reports consultancy fees from Pfizer UK Limited during the conduct of the study and consultancy fees from Pfizer UK Limited outside the submitted work. RC is an employee of Pfizer. Y‐HK reports personal fees from Celltrion during the conduct of the study and personal fees from Chong Kun Dang Pharm, Eisai Korea, Ferring Korea, Janssen Korea, Shire Korea, and Takeda Korea outside the submitted work. MP, OA, MO, AD, SF, OL, and JHC declare no competing interests.	
***Risk of bias***		
**Bias**	**Authors' judgement**	**Support for judgement**
Random sequence generation (selection bias)	Low risk	Quotes: "PPD Bioanalytical Laboratory Services (Bellshill, Scotland, UK) generated the randomisation schedule" so authors contacted and answered that they "generated randomisation code by SAS, then linked to interactive voice response system for randomisation schedule".
Allocation concealment (selection bias)	Low risk	Quotes: "Randomisation codes were not revealed to patients, investigators, or centre personnel, except for predefined unblinded personnel from Celltrion and PPD, until all final clinical data were entered into the database and the database was locked and released for analysis". Authors were asked what the role of the unblinded personnel was and responded "Following our SOP, pre‐defined members were unblinded for development of Week 6 CSR. It included person who was in charge of CSR developing, biostatistician, and who was in charge of regulatory affair (to submit final CSR to regulatory body). Minimum number of personnel was unblinded for reporting purpose. Other than those, people were blinded until study completion. When unblind was needed, all unblind process was approval and logged in writing form".
Blinding of participants and personnel (performance bias) All outcomes	Low risk	Quotes: "Randomisation codes were not revealed to patients, investigators, or centre personnel, except for predefined unblinded personnel from Celltrion and PPD, until all final clinical data were entered into the database and the database was locked and released for analysis". Authors also provided a clarification: "Site personnel and patient could not see their treatment arm fundamentally as they were physically separated from unblinded information."
Blinding of outcome assessment (detection bias) All outcomes	Low risk	Quote: "Randomisation codes were not revealed to patients, investigators, or centre personnel, except for predefined unblinded personnel from Celltrion and PPD, until all final clinical data were entered into the database and the database was locked and released for analysis". Authors provided clarification: "Only preauthorised person was unblinded for reporting purpose at the time of developing clinical study report. Only authorised person could access to unblinded folder. Blinded person could not access to unblinded folder systemically. Other than authorised person could not access to unblinded data (positive regulation scheme. i.e., define unblinded person and prohibit anyone else)".
Incomplete outcome data (attrition bias) All outcomes	Low risk	Attrition accounted for and balanced in all groups, with adequate reasons provided.
Selective reporting (reporting bias)	Low risk	According to trial registration and method section, authors reported relevant endpoint outcomes – clinical response and remission (CDAI scores).
Other bias	Low risk	Baseline characteristics were reported for and balanced in all groups. No other apparent sources of bias.

5‐ASA: 5‐aminosalicylic acid; 6‐MP: 6‐mercaptopurine; AZA: azathioprine; cA2: early name for infliximab; CD: Crohn's disease; CDAI: Crohn's Disease Activity Index; CDEIS: Crohn's Disease Endoscopic Index of Severity; CG: control group; CI: confidence interval; CoI: conflicts of interest; CT‐P13: subcutaneous infliximab; IBDQ: Inflammatory Bowel Disease Questionnaire; IFX: infliximab; IG: intervention group; IQR: interquartile range; IV: intravenous; n: number; NR: not reported; qd: once daily; RCT: randomised controlled trial; SIBDQ: Short Inflammatory Bowel Disease Questionnaire; TNF‐α: tumour necrosis factor‐alpha.

### Characteristics of excluded studies [ordered by study ID]

**Table:**

Study	Reason for exclusion
Baima 2016	Ineligible study design
Bernstein 2002	Ineligible study design
Bernstein 2020	Ineligible study design
Billiet 2016	Ineligible study design
Blesl 2021	Ineligible study design
Blumenstein 2006	Ineligible intervention
Bodini 2018	Ineligible study design
Bortlik 2013	Ineligible study design
Bossuyt 2019	Ineligible study population
Bossuyt 2021	Ineligible study population
Buhl 2020	Ineligible study design
Chaparro 2020	Ineligible study design
Colombel 2019	Ineligible study design
D'Haens 2016	Ineligible study population
EUCTR2008‐006484‐36‐IT	Ineligible study design
EUCTR2010‐018431‐18‐DE	Ineligible study population
EUCTR2011‐003038‐14‐NL	Ineligible study design
EUCTR2021‐000469‐33‐NL	Ineligible intervention (infliximab was given to both groups in the same way and the difference was the additional therapy)
Faegan 2014	Ineligible intervention
Fu 2011	Ineligible study design
Hao 2020	Ineligible study population
Jogensen 2019	Ineligible study population
Khanna 2015	Ineligible study design
Lichtenstein 2002	Ineligible study design
Luna‐Chadid 2003	Ineligible study population
Mantzaris 2004	Ineligible intervention
Mascheretti 2002	Ineligible study population
Narula 2021	Ineligible study design
NCT00004941	Ineligible study population
NCT01442025	Ineligible study design
NCT02883452	Ineligible study population
NCT04835506	Ineligible intervention
Ruemmele 2009	Ineligible study population
Rutgeerts 1999	Ineligible intervention
Rutgeerts 2005	Ineligible study population
Sample 2002	Ineligible study design
Sanchez‐Hernandez 2020	Ineligible study design
Sands 2004c	Ineligible study design
Schroder 2006	Ineligible study population
Sorrentino 2012	Ineligible study population
Syversen 2020a	Ineligible study design
Syversen 2020b	Ineligible study population
Syversen 2021	Ineligible study design
Szymanska 2016	Ineligible study population
Tajiri 2018	Ineligible study population
Yamamoto 2009	Ineligible study population
Yang 2015	Ineligible study design

### Characteristics of ongoing studies [ordered by study ID]

**Table:** KCT0007470

Study name	CHAMELEON Study
Methods	Randomised controlled trial
Participants	100 target sample size
Interventions	"Patients with Crohn's disease will given IV (intravenous) infliximab 5 mg/kg at week 0 and 2. Then, they will be treated with SC (subcutaneous) infliximab every other week. At week 30 patients will be allocated to one of 3 arms according to their response to SC infliximab. [Arm 1] Non‐responder at week 30: switched to infliximab IV 10 mg/kg every 8 weeks Responder at week 30 will be randomly (1:1) allocated to one of the following two arms: [Arm 2] Switched to infliximab IV 5 mg/kg every 8 weeks or [Arm 3] Continued infliximab SC 120 mg every other week"
Outcomes	Primary outcome Non‐inferiority of arm 3 compared with arm 2 in terms of deep remission rate Secondary outcomes Corticosteroid‐free clinical response rate of each arm Non‐inferiority of arm 3 compared with arm 1 in terms of deep remission rate Corticosteroid‐free clinical remission rate of each arm Corticosteroid‐free endoscopic remission rate of each arm Rate of antidrug antibody positivity Corticosteroid‐free complete mucosal healing rate of each arm Corticosteroid‐free clinical biochemical remission rate of each arm Safety evaluation (adverse event, vital signs and laboratory results)
Starting date	30 September 2022
Contact information	Byong Duk Ye Email: bdye@amc.seoul.kr Affiliation: University of Ulsan
Notes	

## Differences between protocol and review

A protocol for this review was published in 2017 from a different author team (Deol 2017). We have updated the methods based on more‐recent Cochrane guidance, and we have amended the outcomes and planned analyses, before commencement of work on the review. We have also updated the background section.

In addition to the databases mentioned in the protocol, we also searched the ClinicalTrials.gov, and the World Health Organization International Clinical Trials Registry Platform. We did not search the Cochrane IBD Group Specialised Register as that is now covered by CENTRAL.

Any planned analyses that are reported in the methods section but not in the results, could not be performed due to lack of sufficient data. We added two subgroup analyses for concomitant immunosuppressant medication use and different disease behaviours, which were not in the original protocol, as we thought they were clinically important. We did not include the subgroup analysis based on baseline characteristics of participants that was mentioned in the protocol, as authors very rarely report result data per characteristic, and subgroup analyses based on these are not possible.

We changed the title of the review to 'Infliximab for medical induction of remission in Crohn's disease' from the protocol title 'Infliximab for induction of remission in Crohn's disease' to clarify that surgical interventions were not considered for this review.
